# Purkinje cell arrival at the Purkinje cell layer orchestrates cerebellar foliation and its temporal sequence

**DOI:** 10.1016/j.isci.2026.116664

**Published:** 2026-09-01

**Authors:** Ryosuke Uchi, Takao Honda, Takuto Kasai, Yuki Hirota, Kazunori Nakajima

**Affiliations:** 1 Department of Anatomy, Keio University School of Medicine, 35 Shinanomachi, Shinjuku-ku, Tokyo 160-8582, Japan; 2 Department of Anatomy, School of Medicine, University of Occupational and Environmental Health, 1-1 Iseigaoka, Yahatanishi-ku, Kitakyushu-shi, Fukuoka 807-8555, Japan

**Keywords:** cerebellar development, foliation, Purkinje cell migration, Reelin signaling, Sonic hedgehog

## Abstract

Cerebellar foliation proceeds in a stereotyped manner, but the developmental mechanisms that regulate its temporal sequence remain unclear. In *reeler* and *yotari*, abnormal Purkinje cell (PC) positioning and foliation defects coexist, yet their causal relationship remains unsolved. Here, using temporally controlled, PC-specific deletion of DAB1, we examined how PC migration relates to foliation. We show that postnatal DAB1 loss disrupts PC positioning and foliation in lobules VI/VII, and that even mild PC displacement from the PC layer (PCL) reduces local proliferation of granule cell precursors (GCPs), while Sonic hedgehog (SHH) supplementation rescues these foliation defects. Furthermore, we find that PC arrival at the PCL differs across lobules, accompanied by lobule-specific peaks in GCP proliferation. These findings identify PC arrival at the PCL as a developmental process that enables effective SHH signaling, thereby promoting foliation, and indicate that spatial differences in PC arrival timing orchestrate the temporal sequence of cerebellar foliation.

## Highlights

• PC arrival at the PCL ensures sufficient SHH delivery to GCPs for foliation
• Even mild PC deviation from the PCL fails to support GCP proliferation and foliation
• Lobule-specific timing of monolayer formation of PCs underlies sequential foliation
• DAB1 is required only until PCs reach the PCL position

## Graphical abstract

**Figure:**
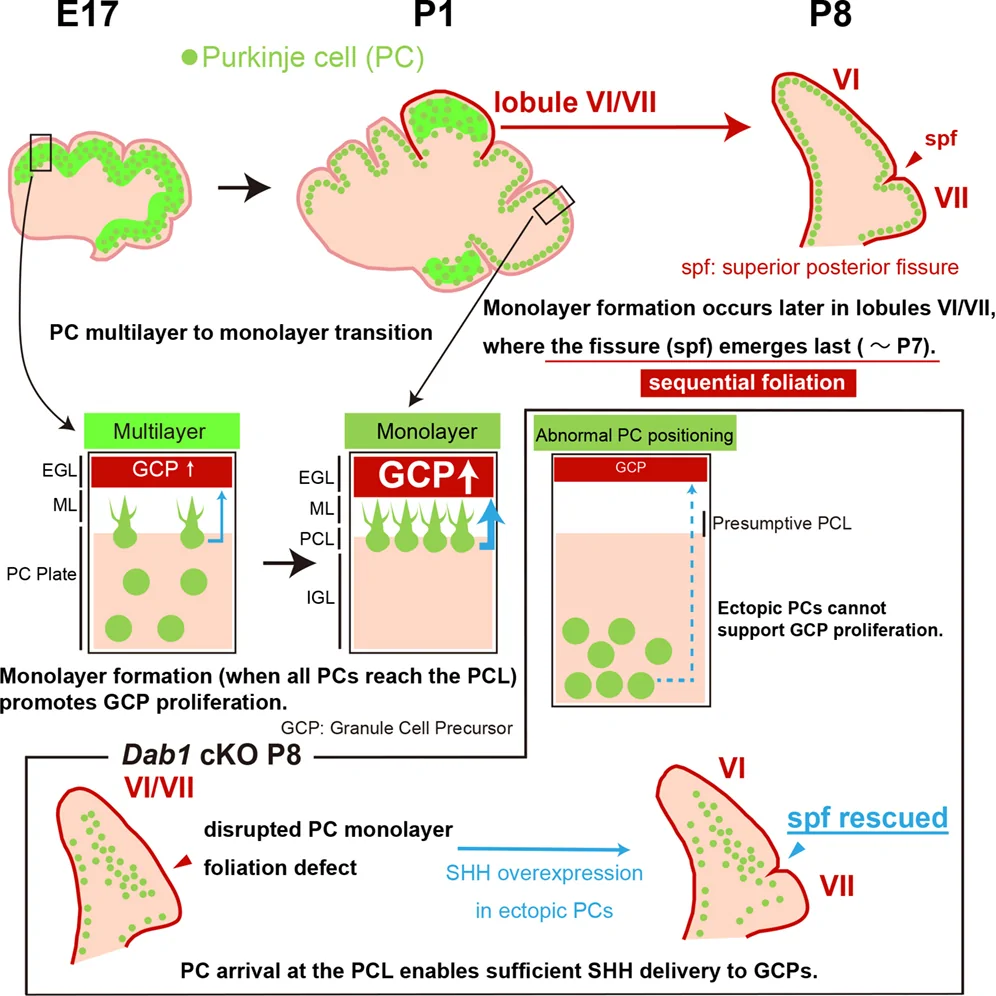

## Teaser

Neuroscience; Cell biology; Developmental biology

## Introduction

Across mammals, the architecture of the brain is characterized by highly organized folds—dramatic in the gyrencephalic neocortex of many species and conserved in the horizontally oriented folia of the cerebellum. In the cerebellum, folia emerge in a highly stereotyped, species-conserved sequence, giving rise to the mature laminated cerebellar cortex—a developmental process collectively referred to as foliation. Yet the developmental principles that generate the precise temporal and spatial pattern of cerebellar folding remain incompletely understood.

Purkinje cells (PCs) and granule cells (GCs) are two essential types of neurons for normal cerebellar development and function. In the mouse developing cerebellum, PCs are generated in the ventricular zone of the cerebellar anlage. They undergo their final mitosis between embryonic day (E) 11 and E13, and subsequently migrate out of the ventricular zone.^1^^,^^2^ After leaving the ventricular zone, PCs migrate toward the cortical surface under the control of Reelin signaling and transiently form a multilayered PC plate (PCP).^3^^,^^4^^,^^5^ Through the early postnatal period, this multilayer gradually resolves into the characteristic monolayer of PCs. As PC migration progresses, cerebellar foliation initiates within the same developmental window. The primary driving force of cerebellar foliation is the proliferation of GC precursors (GCPs) in the external granule layer (EGL). GCP proliferation is strongly promoted by Sonic hedgehog (SHH) secreted from PCs,^6^^,^^7^^,^^8^ and loss of SHH signaling markedly reduces GCP proliferation and results in severe foliation defects.^9^^,^^10^

A particularly intriguing aspect of brain morphogenesis is that folding proceeds in a remarkably sequential manner—both in the gyrencephalic neocortex and the cerebellum. For instance, in the human neocortex, the Sylvian fissure appears first around gestational week (GW) 16,^11^ followed by primary sulci such as the central sulcus by GW 25, and the secondary and tertiary sulci develop during the last 2 months of gestation.^12^ In the mouse cerebellar vermis, major fissures begin to form during late embryogenesis, whereas additional fissures arise only after birth, with those in the central zone (lobules VI/VII) showing the later onset of fissure formation.^13^^,^^14^ To explore the mechanisms underlying this sequential foliation, Legue et al. observed that lobules VI/VII exhibit a later postnatal peak in GCP proliferation,^15^ and Lawton et al. found that lobules destined for secondary folding after birth undergo disproportionate postnatal expansion of the surface area relative to the core.^16^ These findings reveal important correlates of later foliation, but the upstream mechanism that drives the delayed GCP proliferation and surface expansion remains unidentified. The fundamental question of what controls the temporal sequence of fissure formation remains unanswered, and no unifying mechanistic explanation for sequential foliation has yet emerged.

Reelin is a large secreted glycoprotein that plays pivotal roles during brain development, most prominently in controlling neuronal migration and the establishment of laminar architecture.^17^^,^^18^^,^^19^ These developmental actions are mediated through DAB1, the intracellular adapter activated downstream of the Reelin receptors LRP8 (ApoER2) and VLDLR.^20^^,^^21^^,^^22^^,^^23^ During cerebellar development, *Reelin* expression is first detected in rhombic lip-derived neurons within the nuclear transitory zone and subsequently in GCPs of the EGL,^24^^,^^25^^,^^26^ whereas *Dab1* is expressed predominantly in PCs.^27^ Mouse mutants with disrupted Reelin signaling, such as the *reeler and yotari* (a *Dab1* null mutant), commonly exhibit ectopic PCs that remain deeply positioned as well as a marked foliation defect.^4^^,^^28^^,^^29^^,^^30^ However, it remains unknown how aberrant PC positioning influences the initiation and progression of fissure formation, to what extent PC mispositioning perturbs GCP proliferation, and whether the foliation defect in *Reelin*/*Dab1* mutant cerebella can be explained solely by abnormal PC migration. Moreover, the precise developmental window during which DAB1 is required for PC migration has not been defined. Consequently, a unifying mechanistic link that connects Reelin signaling, PC migration, and cerebellar foliation remains unclear.

In the present study, we set out to clarify how PC migration governs cerebellar foliation and to uncover the stage-specific contribution of *Dab1* to this process. By establishing a temporally precise *Dab1* conditional knockout (cKO) system, we revealed two key mechanisms. First, PC arrival at the PC layer (PCL) is a critical regulatory step that controls the availability of PC-derived cues required for GCP expansion. Second, intrinsic differences in the timing of PC arrival across lobules provide an upstream mechanism that can account for sequential fissure formation. Together, these findings establish a unifying framework linking Reelin-DAB1 signaling, PC migration dynamics, and the temporal patterning of cerebellar foliation.

## Results

### Postnatal loss of *Dab1* locally disrupts PC alignment and foliation

Because the severe foliation defects in *reeler* and *yotari* mice arise from major fissures formed during the embryonic stage,^31^ the role of the Reelin-DAB1 pathway in postnatal formation and remodeling of cerebellar fissures remains unclear.

To address this question, we ablated *Dab1* after birth using adeno-associated virus (AAV)-mediated Cre expression. AAV-CAG-EGFP-P2A-Cre was injected bilaterally into the lateral ventricles of *Dab1*^*fl/fl*^ and *Dab1*^*+/fl*^ littermates (*Dab1 flox* mice) at postnatal day (P) 1, and the cerebella were fixed at P8. EGFP served as a marker for Cre-transfected cells. Immunostaining revealed that the majority of EGFP-positive cells were Calbindin-positive PCs,^32^ while a small population consisted of BLBP-positive astrocytes^33^ in the internal granule layer (IGL) and Nestin-positive Bergmann glia (BG)^34^^,^^35^ (Figures S1A–S1C). Although the number of transduced PCs varied among lobules, presumably due to local differences in viral spread, overall transduction was widespread throughout the cerebellar cortex (Figure S1D). Morphological analysis revealed that in *Dab1*^*fl/fl*^ mice, overall cerebellar architecture was grossly normal, but the superior posterior fissure (spf) between lobules VI and VII was selectively and markedly shallow and in some cases even absent (Figures S1D and S1E). Calbindin immunostaining showed that PCs in *Dab1*^*+/fl*^ control formed a continuous monolayer, whereas those in *Dab1*^*fl/fl*^ mice exhibited disrupted layering and ectopic PCs within the IGL (Figure S1F). The degree of PC disorganization varied among lobules and was most prominent in lobules VI/VII and X (Figure S1G).

**Figure 1:**
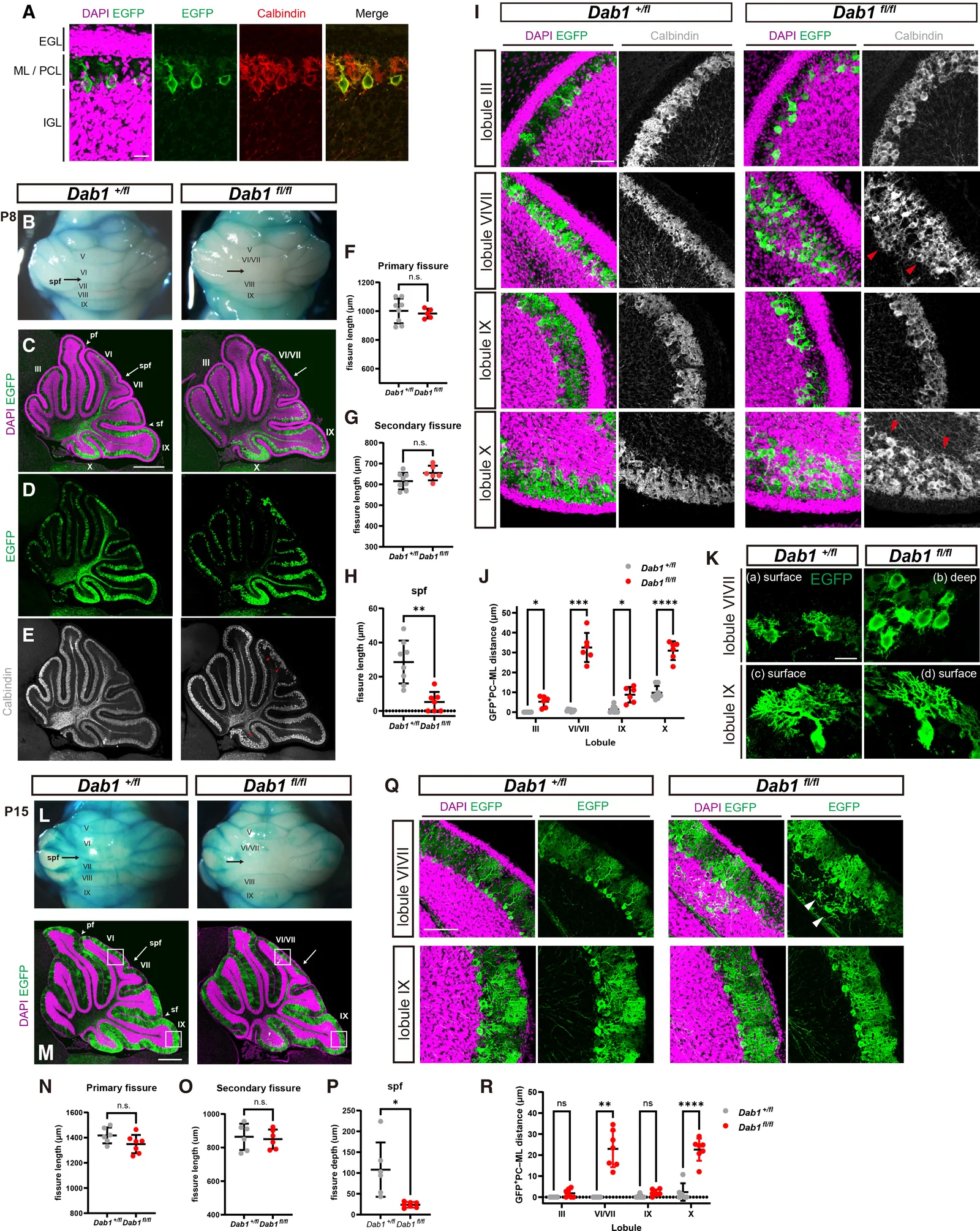
Postnatal loss of DAB1 in PCs disrupts PC layer organization and selectively impairs the formation of superior posterior fissure (A) Sagittal cerebellar sections from *Dab1*^*+/fl*^ mice immunostained for EGFP (green) and Calbindin (red) with DAPI (magenta). (B) Stereomicroscopic images of the cerebellum. Superior posterior fissure (spf) is indicated by an arrow. (C–E) Sagittal sections of the cerebellar vermis. (C) The primary fissure (pf), secondary fissure (sf), and spf are indicated by arrowheads and an arrow, respectively. (D) EGFP signal. (E) Calbindin immunostaining (white); disrupted Purkinje cell (PC) layer organization in lobules VI/VII and X is indicated by red arrowheads. (F–H) Quantification of fissure depth of pf, sf, and spf in *Dab1*^*+/fl*^ and *Dab1*^*fl/fl*^ mice. (I) Higher-magnification images of lobules III, VI/VII, IX, and X, showing PC layer disruption in *Dab1*^*fl/fl*^ mice (red arrowheads). (J) Quantification of the distance between EGFP-positive PCs at the folial surface and the molecular layer (ML). (K) Higher-magnification EGFP images of lobules. (L) Stereomicroscopic images of the cerebellum. (M) Sagittal vermis sections, showing the spf (arrows). Arrowheads in *Dab1*^*fl/fl*^ indicate ectopic PCs. (N–P) Quantification of fissure depth of pf, sf, and spf. (Q) Higher-magnification images of lobules, showing PC layer disruption in *Dab1*^*fl/fl*^ mice (arrowheads). (R) Quantification of the distance between EGFP-positive PCs at the folial surface and the ML. *Dab1 flox* mice were injected with AAV-L7-EGFP-P2A-Cre virus at postnatal day 1 (P1), and cerebella were analyzed at P8 (A–K) or P15 (L–R). Scale bars, 20 μm (A and K), 100 μm (I), 500 μm (C–E and M), and 200 μm (Q). Data are shown as mean ± SD. *n* = 6–9 mice per genotype at both P8 and P15, except for the quantification of pf depth at P8 in *Dab1*^*fl/fl*^ mice (*n* = 5). Fissure depth was measured on midline sagittal sections; for spf, four sections per animal were analyzed and the maximal depth was used for quantification (see STAR Methods for details). Statistical significance was assessed using unpaired two-tailed *t* tests (F–H and N–P). EGFP-positive PC–ML distance across lobules was analyzed using two-way ANOVA with Sidák’s multiple comparisons test (J and R). EGL, external granule layer; PCL, Purkinje cell layer; ML, molecular layer; IGL, internal granule layer.

To determine whether these abnormalities result specifically from loss of *Dab1* in PCs, we then generated an AAV expressing EGFP and Cre under the PC-specific L7 promoter^36^ (AAV-L7-EGFP-P2A-Cre) and injected it into *Dab1 flox* mice at P1. Nearly all EGFP-positive cells were Calbindin-positive PCs, confirming cell-type specificity (Figure 1A). Stereomicroscopic observation revealed that the overall cerebellar architecture appeared intact, while the spf was unclear in *Dab1*^*fl/fl*^ mice (Figure 1B). Morphological and quantitative analysis revealed that major fissures such as the primary (pf) and secondary fissure (sf) were unaffected, whereas the spf was markedly reduced or almost absent in *Dab1*^*fl/fl*^ mice (Figures 1C–1H). Calbindin immunostaining showed that, in control mice, PCs formed a continuous monolayer, whereas in *Dab1*^*fl/fl*^ mice, the PCL was disrupted, and ectopic PCs were embedded within the IGL (Figures 1E and 1I, arrowheads). The severity of PC disorganization varied among lobules and was most evident in lobules VI/VII and X (Figures 1I and 1J). Ectopic PCs in *Dab1*^*fl/fl*^ mice have markedly underdeveloped dendrites compared with PCs that successfully reached the PCL in control mice (Figure 1K (a), (b)). In contrast, PCs that reached above the presumptive IGL, where the PCs should naturally exist, in *Dab1*^*fl/fl*^ mice exhibited mature morphology similar to that of the controls (Figure 1K (c), (d)).

The phenotypes observed in the PC-specific knockout (KO) were identical to those seen in CAG-Cre-mediated *Dab1* deletion, confirming that the defects in local foliation and layering arise from the loss of *Dab1* specifically within PCs.

To evaluate whether these localized effects persisted at later stages, *Dab1* cKO mice injected at P1 were analyzed at P15. The spf remained selectively reduced in depth in *Dab1*^*fl/fl*^ cerebella, while other fissures were unaffected (Figure 1L–1P). Abnormal PC positioning was again observed, and, as at P8, confined to lobules VI/VII and X (Figures 1M, 1Q, and 1R). Ectopic PCs remained immature, whereas those that reached near the surface in *Dab1*^*fl/fl*^ mice developed dendrites comparable to those of the control (Figure 1Q), suggesting that PC maturation is triggered by arrival at the presumptive PCL position itself, and that DAB1 may not directly regulate the intrinsic maturation program.

Together, these results indicate that postnatal DAB1 in PCs is required for proper, lobule-specific PC positioning and the corresponding fissure development.

### DAB1 is only required until PCs reach the presumptive PCL position

**Figure 2:**
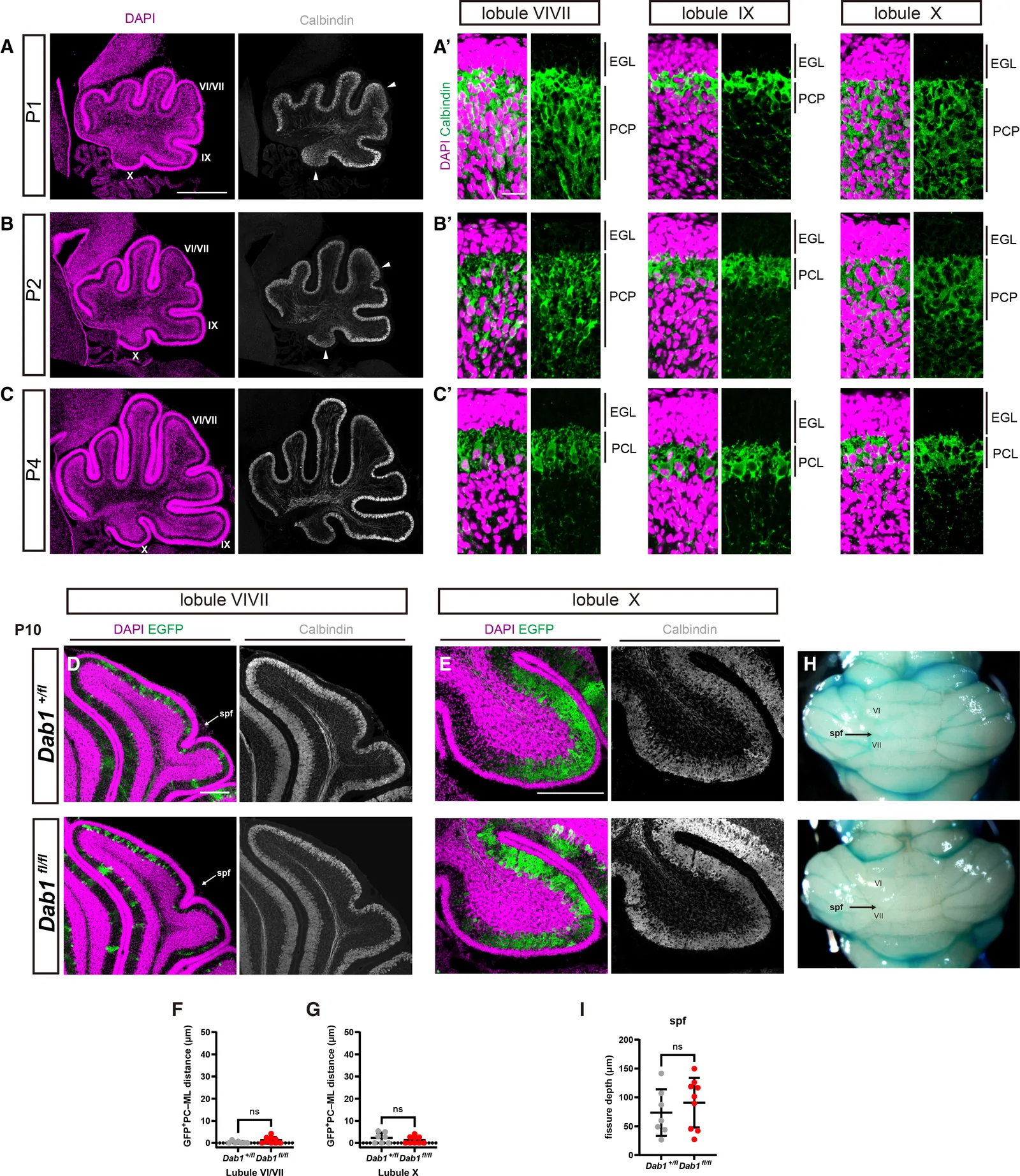
PC arrival at the presumptive PCL position differs across lobules, and *Dab1* deletion after PC monolayer formation does not affect PC position or foliation (A–C) Sagittal cerebellar sections from wild-type mice at P1 (A), P2 (B), and P4 (C) immunostained for Calbindin (white or green) with DAPI (magenta). White arrowheads (A and B) indicate multilayered PCs. (A′–C′) Higher-magnification views showing lobules VI/VII, IX, and X. (D–I) *Dab1 flox* mice were injected with AAV-L7-EGFP-P2A-Cre virus at P4, and cerebella were analyzed at P10. (D and E) Sagittal cerebellar sections showing lobule VI/VII (D) and X (E). EGFP is shown in green and Calbindin in white. Arrowheads (D) indicate the spf. (F and G) Quantification of the distance between EGFP-positive PCs at the folial surface and the ML in *Dab1*^*+/fl*^ and *Dab1*^*fl/fl*^. (H) Stereomicroscopic images of the cerebellum. Superior posterior fissure (spf) is indicated by arrows. (I) Quantification of spf depth in *Dab1*^*+/fl*^ and *Dab1*^*fl/fl*^. Scale bars, 200 μm (A–C), 20 μm (A′–C′), and 100 μm (D and E). PC–ML distance data are shown as mean ± SD (F and G), and spf depth are shown as median with interquartile range (I). *n* = 7–9 mice per genotype. Statistical significance was assessed using unpaired two-tailed t tests for PC–ML distance (F and G) and the Mann-Whitney U test for fissure depth (I). PCP, Purkinje cell plate.

Next we addressed why postnatal loss of *Dab1* caused pronounced PC disorganization specifically in lobules VI/VII and X. Because PC migratory dynamics are known to vary among lobules,^37^^,^^38^ we hypothesized that region-specific patterns of migration might underlie this selective disruption. To explore this, we examined the migratory behavior of PCs over developmental time in wild-type cerebella. In most lobules—illustrated by lobule IX—all PCs had already reached the presumptive PCL position and formed a continuous monolayer by P1–P2 (Figures 2A, 2A′, 2B, and 2B′). In contrast, in lobules VI/VII and X, PCs remained in a multilayer at P1-P2 and reached the presumptive PCL to form a monolayer only around P4 (Figures 2A–2C and 2A′–2C′). These observations suggest that DAB1 in PCs is required for their migration until PCs reach the presumptive PCL position. Since PCs in most lobules (such as lobules III and IX) had already arrived at the presumptive PCL by the time of AAV injection, the PCs in those lobules would be largely unaffected by *Dab1* deletion.

To test this hypothesis, we injected AAV-L7-EGFP-P2A-Cre into *Dab1 flox* mice at P4, when almost all PCs had already reached near the surface and established a monolayer. As expected, abnormal PC positioning was rarely observed in lobules VI/VII and X, or in any lobules (Figures 2D–2G). The spf also developed normally (Figures 2D and H), with no significant difference in depth between *Dab1*^*fl/fl*^ and *Dab1*^*+/fl*^ littermates (Figure 2I).

These results support the hypothesis that DAB1is required for PC migration only until the monolayer formation, and demonstrate that once PCs properly form a monolayer, normal foliation can proceed even in the absence of DAB1. When considered together with the findings from the Figure 1 experiments—in which foliation defects occur only in lobules VI/VII where *Dab1*-deficient PCs fail to reach the presumptive PCL position, while regions containing *Dab1*-deficient PCs that nevertheless reach the presumptive PCL position form normal fissures (e.g., the pf and sf)—these results indicate that DAB1 promotes foliation through establishing correct PC positioning, not by directing PC-intrinsic developmental programs.

### Even mild displacement of PCs from the presumptive PCL position impairs local GCP proliferation

To investigate why impaired PC migration leads to local foliation defects, we considered the possibility that GCP proliferation might be affected locally. Indeed, Smeyne et al., using L7-CD toxin transgenic mice, showed that regions lacking PCs exhibit a thinner EGL, whereas areas retaining PCs display a thicker EGL, suggesting that the presence of PCs supports GCP expansion.^39^ However, whether the position of PCs relative to the pial surface modulates local GCP proliferation has remained unclear.

**Figure 3:**
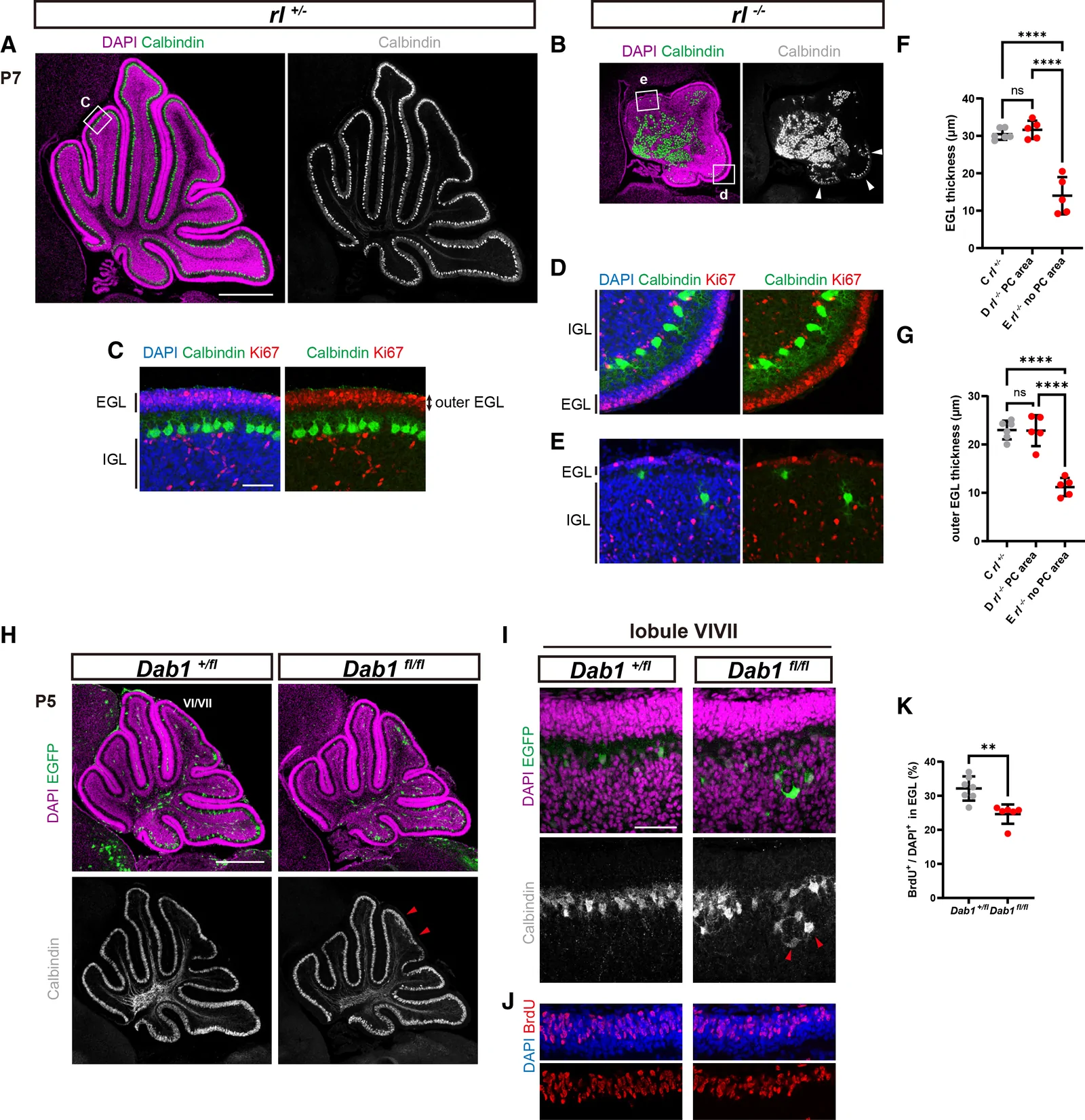
PC displacement from the PCL impairs granule cell precursor proliferation in *reeler* and *Dab1* cKO cerebella (A and B) Sagittal cerebellar sections from *rl*^*+/−*^ (A) and *rl*^−/−^ (B) mice at P7 immunostained for Calbindin (green) with DAPI (magenta). Calbindin signal alone is also shown in white. Arrowheads in *rl*^−/−^ mice (B) indicate a small subset of PCs that have reached the presumptive PCL position. (C–E) Higher-magnification views of the boxed regions indicated in A and B immunostained for Calbindin (green) and Ki67 (red) with DAPI (blue). The outer EGL is indicated by a double-headed arrow. (F and G) Quantification of the total EGL thickness (F) and the outer EGL thickness (G), comparing *rl*^*±*^ regions (as shown in C), *rl*^−/−^ regions containing PCs that have reached the presumptive PCL position (D), and *rl*^−/−^ regions lacking PCs that have reached the presumptive PCL position (E). Regions containing three or more PCs that have reached the presumptive PCL position within a 150-μm-wide segment were classified as D group, whereas regions containing one or fewer such cells were classified as E group. (H–K) *Dab1 flox* mice were injected with AAV-L7-EGFP-P2A-Cre virus at P1, and cerebella were analyzed at P5 1 h after BrdU injection. (H) Sagittal cerebellar sections, immunostained for EGFP (green) and Calbindin (white) with DAPI (magenta). Arrowheads indicate disorganized PC alignment in *Dab1*^*fl/fl*^ mice. (I) Higher-magnification view of lobules VI/VII. Arrowheads indicate ectopically positioned PCs in the IGL. (J) Immunostaining for BrdU (red) with DAPI (blue) in the EGL. (K) Quantification of the ratio of BrdU-positive cells to DAPI-positive cells in the EGL. Scale bars, 500 μm (A, B, and H), 100 μm (C–E), and 50 μm (I and J). Data are shown as mean ± SD. Sample sizes were as follows: *reeler* mice, *rl*^*+/−*^ (*n* = 6) and *rl*^−/−^ (*n* = 5); *Dab1 flox* mice, *Dab1*^*+/fl*^ (*n* = 7) and *Dab1*^*fl/fl*^ (*n* = 6). For EGL thickness (F and G), three points within a single section were measured and averaged per animal. For BrdU/DAPI quantification (K), one section per animal was analyzed, and cells were counted within the outer 150 μm of the EGL. Statistical significance was assessed using one-way ANOVA followed by Tukey’s multiple comparisons test for the EGL thickness in *reeler* mice (F and G) and unpaired two-tailed *t* tests for BrdU^+^/DAPI^+^ cells (K).

To address this, we examined cerebellar sections from *reeler* (*rl*^−/−^) mice. In *rl* ^*±*^ mice at P7, PCs were aligned near the cortical surface (just above the IGL), and the EGL appeared uniformly thick across lobules (Figure 3A). In contrast, in *rl*^−/−^ mice, most PCs remained deeply located, while a small subset had reached near the cortical surface (arrowheads in Figure 3B). The EGL was visibly thicker specifically in regions containing surface PCs than in regions where PCs remained in deeper positions (Figures 3C–3E). Consistent with these observations, quantitative analysis revealed that the EGL and its Ki67^+^ outer proliferative zone (outer EGL) were significantly thicker in areas where PCs had reached near the cortical surface than in those devoid of surface PCs in *rl*^−/−^ mice, with the thickness in PC-rich areas being comparable to that in *rl* ^*±*^ mice (Figures 3F and 3G). These findings suggest that abnormal positioning of PCs may lead to impaired GCP proliferation. We therefore hypothesized that reduced GCP proliferation could underlie the local foliation defects observed in *Dab1* cKO mice.

To test this possibility, we assessed GCP proliferation at P5 in *Dab1* cKO mice, a stage preceding foliation defects, using 5-bromo-2′-deoxyuridine (BrdU) pulse labeling. AAV-CAG-EGFP-P2A-Cre was injected, and the cerebella were fixed 1 h after BrdU administration at P5. Consistent with the results shown in Figure 1, abnormal PC positioning in the IGL was observed in lobules VI/VII (Figures 3H and 3I). Quantification of BrdU^+^/DAPI^+^ cells in the EGL of lobules VI/VII revealed a significant reduction in *Dab1* ^*fl/fl*^ mice compared with *Dab1* ^*+/fl*^ controls (Figures 3J and 3K), suggesting that even mild displacement of PCs from their normal position can compromise local GCP proliferation.

### SHH supplementation restores foliation defects caused by impaired PC migration

Since PC-derived SHH is known to promote GCP proliferation, we reasoned that the decreased GCP proliferation in regions with ectopic PCs in *Dab1* ^*fl/fl*^ mice was attributable to reduced SHH supply to the EGL. To test whether SHH supplementation could restore foliation in lobules VI/VII, we expressed SHH specifically in PCs by co-injecting AAV-L7-EGFP-P2A-Cre (“AAV (L7)”) together with AAV-CAG-DIO-*Shh* (“AAV (Shh)”) into *Dab1 flox* mice. We first examined whether forced SHH expression in PCs affects cerebellar morphology under normal conditions. To this end, AAV (L7) or a combination of AAV (L7) and AAV (Shh) was injected into *Dab1* ^*+/fl*^ mice at P1 and the cerebella were collected at P8 (Figure S2A). Immunostaining confirmed strong SHH immunoreactivity in a subset of EGFP-positive PCs, while PCs in both groups maintained a normal monolayer arrangement (Figure S2B). The overall cerebellar architecture—including the pf, sf, and spf—was indistinguishable between these groups (Figures S2A–S2F). These results indicate that the level of SHH expression achieved by this system is physiologically appropriate.

**Figure 4:**
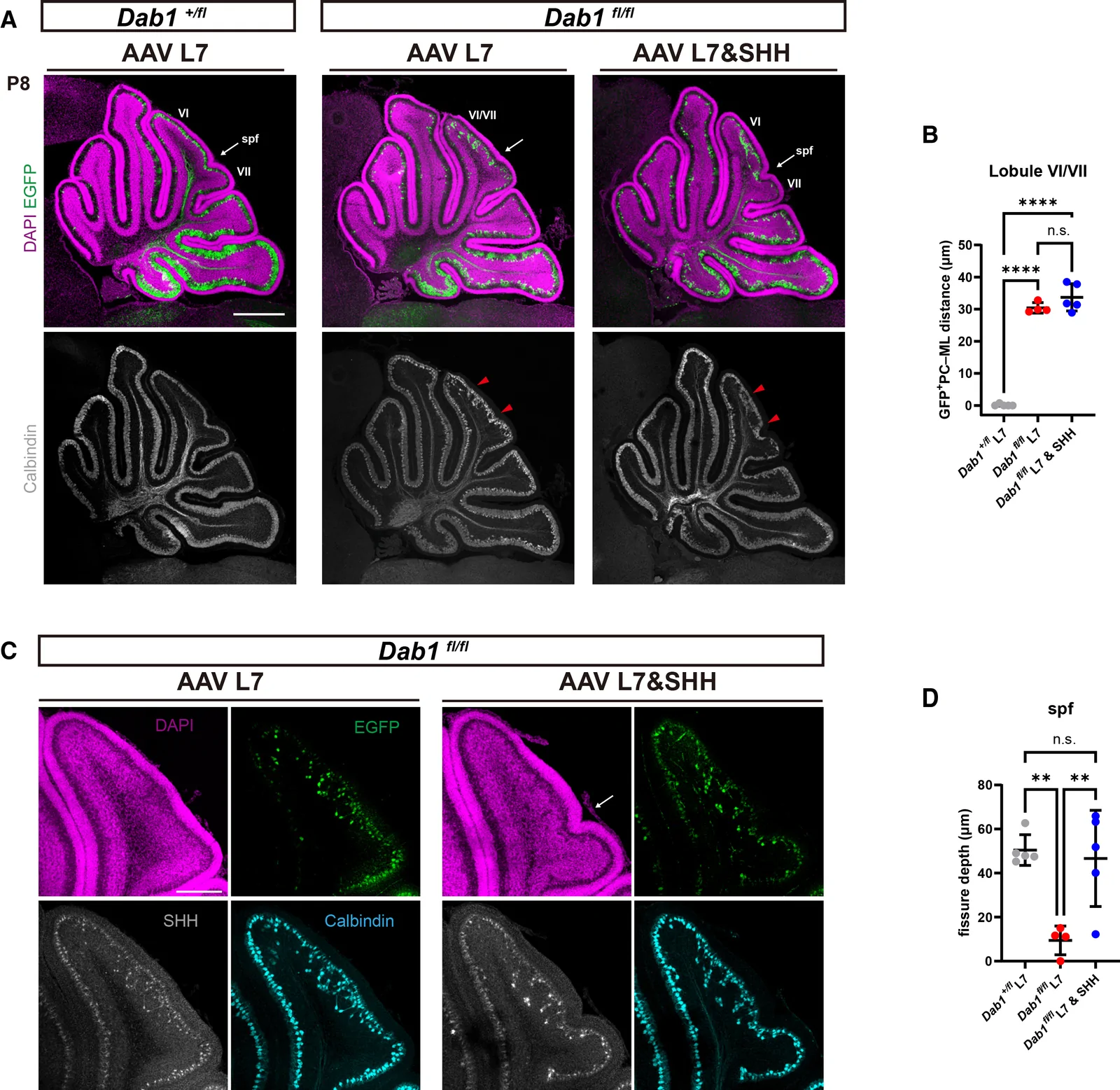
SHH expression rescues cerebellar foliation defects despite persistent PC mispositioning (A) Sagittal cerebellar sections immunostained for EGFP (green) and Calbindin (white) with DAPI (magenta). Arrows indicate the spf. In the Calbindin images, arrowheads mark PC layer disruption. (B) Quantification of the distance between EGFP-positive PCs at the folial surface and the ML. (C) Higher-magnification view of lobules VI/VII, immunostained for EGFP (green), SHH (white), and Calbindin (cyan) with DAPI (magenta). Arrow indicates the spf. (D) Quantification of the spf depth. *Dab1 flox* mice were injected with AAV at P1 and cerebella were analyzed at P8. Three experimental groups are shown: (*Dab1*^*+/fl*^ L7) *Dab1*^*+/fl*^ mice injected with AAV-L7-EGFP-P2A-Cre (control); (*Dab1*^*fl/fl*^ L7) *Dab1*^*fl/fl*^ mice injected with AAV-L7-EGFP-P2A-Cre (*Dab1* cKO); and (*Dab1*^*fl/fl*^ L7 &SHH) *Dab1*^*fl/fl*^ mice injected with AAV-L7-EGFP-P2A-Cre and AAV-CAG-DIO-SHH (*Dab1* cKO and SHH expression). Scale bars, 500 μm (A) and 200 μm (C). Data are shown as mean ± SD. *n* = 4–6 mice per group. For fissure length of the spf, four sections per animal were analyzed and the maximal depth was used for quantification. Statistical significance was assessed using one-way ANOVA followed by Tukey’s multiple comparisons test (B and D).

Then we investigated whether SHH supplementation could restore the foliation defects in *Dab1* cKO. Three experimental groups were analyzed: *Dab1*^*+/fl*^ mice injected with AAV (L7) (Ctrl), *Dab1*^*fl/fl*^ mice injected with AAV (L7) (KO), and *Dab1*^*fl/fl*^ mice co-injected with AAV (L7) and AAV (Shh) (KO + rescue). As in previous experiments, abnormal PC positioning was observed in lobules VI/VII of both KO and KO + rescue mice, with the same degrees of displacement (Figures 4A and 4B). In addition, we did not observe obvious differences in PC morphology between KO and KO + rescue mice. Notably, however, the spf in the rescue group was restored to a depth comparable to that of *Dab1* ^*+/fl*^ controls (Figures 4C and 4D). These results indicate that SHH supplementation at a dose that does not affect foliation in control cerebella can compensate for the local foliation defects caused by impaired PC migration. Moreover, restored fissure occurred at the same location as in *Dab1*^*+/fl*^ controls despite the PC misalignment, indicating that precise PC positioning does not determine fissure position, although normal PC positioning is required for the sufficient SHH delivery to GCPs.

### PC positioning, not SHH production, determines SHH delivery

**Figure 5:**
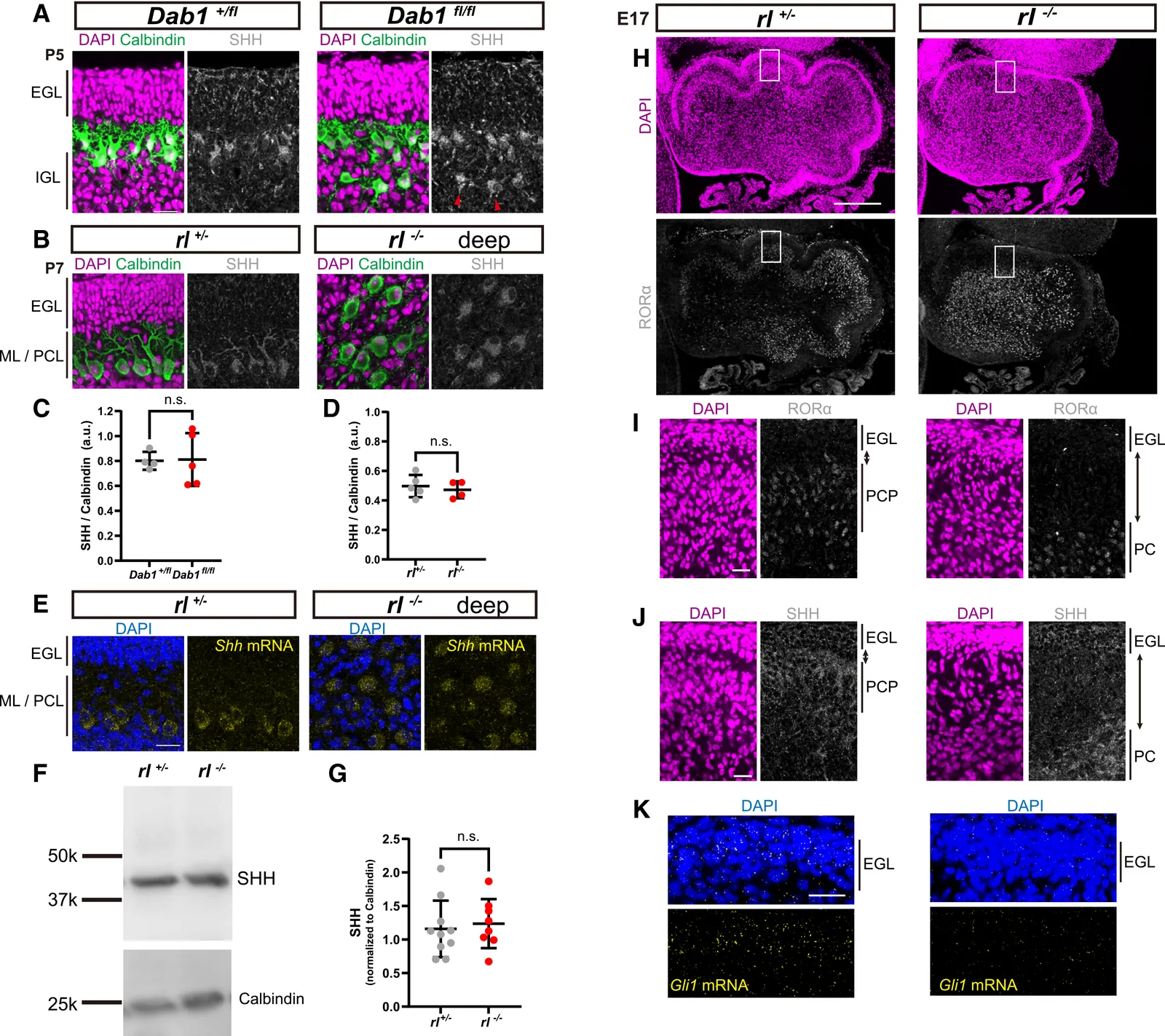
PC mispositioning does not alter SHH production but reduces SHH signaling in the EGL (A and B) Sagittal cerebellar sections from *Dab1 flox* mice injected with AAV-CAG-EGFP-P2A-Cre virus at P1 and analyzed at P5 (lobules VI/VII) (A) and sections from heterozygous and homozygous *reeler* mice at P7 (B), immunostained for Calbindin (green) and SHH (white) with DAPI (magenta). Ectopic PCs are indicated by red arrowheads (A). PCs at the presumptive PCL position in *rl*^*±*^ mice are compared with deeply located PCs in *rl*^−/−^ mice (B). (C and D) Quantification of SHH fluorescence normalized to Calbindin in individual PCs corresponding to (A) and (B), respectively. Values are shown in arbitrary units (a.u.). (E) *In situ* hybridization for *Shh* in sagittal cerebellar sections from *reeler* mice at P7. *Shh* transcripts are shown in yellow with DAPI (blue). (F) Western blot analysis of SHH and Calbindin in cerebellar lysates from *reeler* mice at P7. (G) The SHH/Calbindin signal ratio was quantified in *rl*^*±*^ and *rl*^−/−^ mice. (H–K) Sagittal cerebellar sections from *reeler* mice at E17. (H) Sections immunostained for RORα (white) with DAPI (magenta). (I) Higher-magnification views of the boxed regions in (H). (J)Sections from the anterior lobule immunostained for SHH (white) with DAPI (magenta). The distance between the EGL and PCs is indicated by double-headed arrows (I and J). (K) *In situ* hybridization for *Gli1* in the EGL of anterior lobules. *Gli1* transcripts are shown in yellow with DAPI (blue). Scale bars, 20 μm (A, B, E, and I–K) and 200 μm (H). Data are shown as mean ± SD. *Dab1*^*+/fl*^ (*n* = 4) and *Dab1*^*fl/fl*^ (*n* = 5) (C), *n* = 5 (*rl*^*+/−*^) and 4 (*rl*^−/−^) (D), and *n* = 10 (*rl*^*+/−*^) and 8 (*rl*^−/−^) mice (G). For SHH/Calbindin ratio (C and D), three sections per animal were analyzed, and three PCs were quantified per section. Statistical significance was assessed using unpaired two-tailed *t* tests (C, D, and G). PCP, Purkinje cell plate.

Our findings so far suggest that PCs must reach the presumptive PCL position to sufficiently deliver SHH to the EGL. We next asked whether impaired SHH signaling in regions with ectopic PCs reflects reduced SHH production itself, or whether SHH is normally produced but fails to reach the EGL. SHH immunostaining in *Dab1 flox* cerebella at P5 revealed comparable signal intensity between properly positioned PCs in *Dab1*^*+/fl*^ mice and ectopic PCs within the IGL in *Dab1*^*fl/fl*^ mice (Figure 5A), which was confirmed by quantification (Figure 5C). Similar comparisons in *reeler* cerebella at P7 showed no significant difference in SHH protein levels between surface PCs in *rl* ^*±*^ mice and deeply located PCs in *rl*^−/−^ mice (Figures 5B and 5D). Furthermore, *in situ* hybridization in *reeler* mice showed no obvious difference in *Shh* mRNA expression between genotypes (Figure 5E).

Because PCs are the sole source of SHH in the cerebellum, the protein levels detected in cerebellar lysates reflect SHH production in PCs.^6^^,^^8^ Western blot analysis of cerebellar lysates from *rl* ^*±*^ and *rl*^−/−^ mice at P7 showed comparable levels of the SHH precursor (45 kDa) between genotypes (Figure 5F). Quantification of the SHH band intensity, normalized to Calbindin, confirmed no significant difference in SHH levels between the two genotypes (Figure 5G). These findings suggest that SHH expression is cell-intrinsic and independent of Reelin signaling and the positional state of PCs.

To further assess the effect of PC position on SHH signaling in the EGL, we analyzed embryonic *reeler* cerebella, in which PC location can be clearly distinguished from that of normal mice. PC position was visualized using an anti-RORα antibody, which primarily labels PCs at embryonic stages.^40^^,^^41^ PC positions already show differences between genotypes at E15, with some PCs in *rl*^*±*^ mice beginning to form a multilayered PCP (Figure S3). By E17, PCs in *rl* ^*±*^ mice had moved further toward the cortical surface and formed a clear PCP, whereas PCs in *rl*^−/−^ mice remained deeply located (Figures 5H and 5I). Consistent with our postnatal analyses, SHH immunoreactivity was similar between surface-located PCs in *rl* ^*±*^ mice and deeply located PCs in *rl*^−/−^ mice (Figure 5J). However, *Gli1* expression in the EGL, reflecting SHH pathway activity in GCPs, showed a clear contrast: *Gli1* signals were strong in regions above the surface PCs in *rl* ^*±*^ mice, whereas the EGL overlying deeply located PCs in *rl*^−/−^ mice exhibited markedly reduced *Gli1* signals (Figure 5K).

Together, these findings indicate that the spatial position of PCs—specifically their proximity to the EGL or their appropriate localization to the presumptive PCL—acts as a critical determinant of SHH delivery and downstream signaling in the EGL, even though SHH production remains unchanged.

### The late peak of GCP proliferation coincides with delayed spf formation

**Figure 6:**
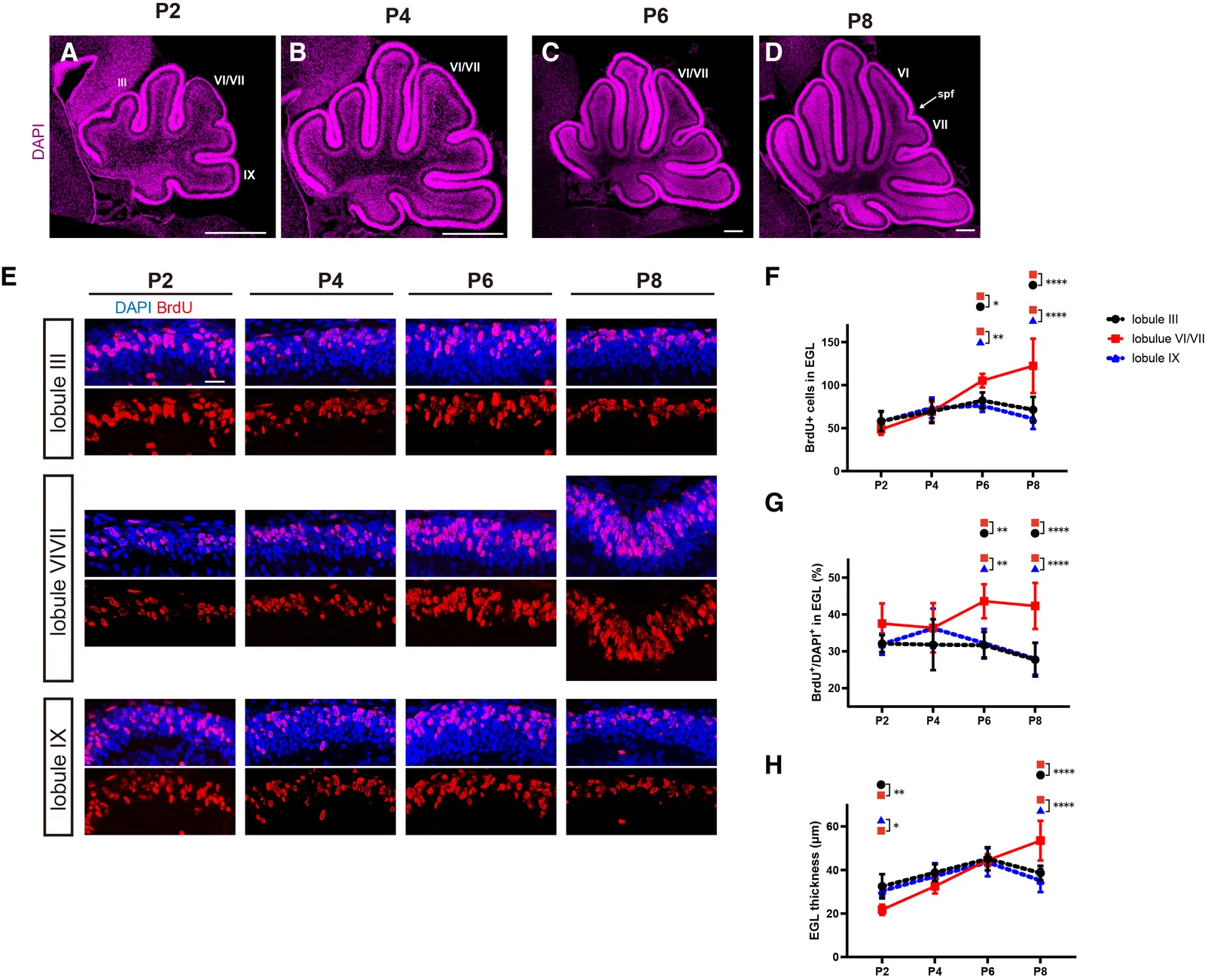
Temporal peak of GCP proliferation occurs later in lobules VI/VII (A–D) Sagittal cerebellar sections from wild-type mice at P2, 4, 6, and 8. The superior posterior fissure (spf) is indicated by an arrow (D). (E–H) Wild-type cerebella were fixed 1 h after BrdU injection at P2, 4, 6, or 8. (E) Immunostaining of the EGL in lobules III, VI/VII, and IX, showing DAPI (blue) and BrdU (red). (F and G) Quantification of the number of BrdU-positive cells (F) and the ratio of BrdU-positive cells to DAPI-positive cells (G) within the outer 150 μm of the EGL in lobules III,VI/VII, and IX at each developmental stage. (H) Quantification of the thickness of the EGL in the indicated lobules at each developmental stage. Scale bars, 200 μm (A–D) and 20 μm (E). Data are shown as mean ± SD. Sample sizes were as follows: *n* = 6 (P2), *n* = 6 (P4), *n* = 5 (P6), and *n* = 6 (P8) mice. For EGL thickness (H), three points within a single section were measured and averaged per animal. For BrdU/DAPI quantification (F and G), one section per animal was analyzed, and cells were counted within the outer 150 μm of the EGL. Statistical analysis was performed using two-way ANOVA followed by Tukey’s multiple comparisons test, comparing lobules within each developmental stage.

We next focused on the spf, the fissure selectively lost in *Dab1* cKO mice (Figure 1). Examination of wild-type cerebella (C57BL/6N) across postnatal stages revealed that most fissures were already formed by P2, whereas the spf emerged only around P7–8 (Figures 6A–6D). This difference in developmental timing prompted us to examine whether the timing of GCP proliferation differs between lobules VI/VII and other lobules. To address this, we administered BrdU in wild-type mice at P2, P4, P6, and P8, fixed cerebella 1 h later, and quantified BrdU^+^ cells in the EGL. In lobules III and IX, BrdU^+^ cells increased modestly from P2 to P6 and declined by P8. In contrast, lobules VI/VII showed a distinct surge in BrdU^+^ cells at P6, followed by a continued increase through P8 (Figures 6E and 6F). The BrdU^+^/DAPI^+^ fractions exhibited a similar trend: whereas lobules III and IX showed a decline by P8, lobules VI/VII maintained highly proliferative activity (Figure 6G). EGL thickness followed the same pattern, increasing steadily in lobules VI/VII while peaking earlier in other lobules (Figure 6H). These region-specific dynamics are consistent with the earlier work by Legue et al.,^15^ who demonstrated a similarly later peak of GCP proliferation in lobules VI/VII, using a different mouse strain.

Together, these observations indicate that lobules VI/VII exhibit a uniquely late rise in GCP proliferation, corresponding with the late formation of the spf. Notably, this later proliferative peak aligns with two features revealed in earlier figures: PCs in lobules VI/VII reach the presumptive PCL position later than PCs in other lobules (Figure 2), and PCs must reach the PCL position to effectively deliver SHH and drive GCP proliferation (Figures 3, 4, and 5). Integrating these findings, we propose that the timing of PC arrival at the presumptive PCL position may help determine when GCP proliferation intensifies and, consequently, when individual fissures form.

### Local delays in PC migration result in region-specific foliation defect during embryonic development

**Figure 7:**
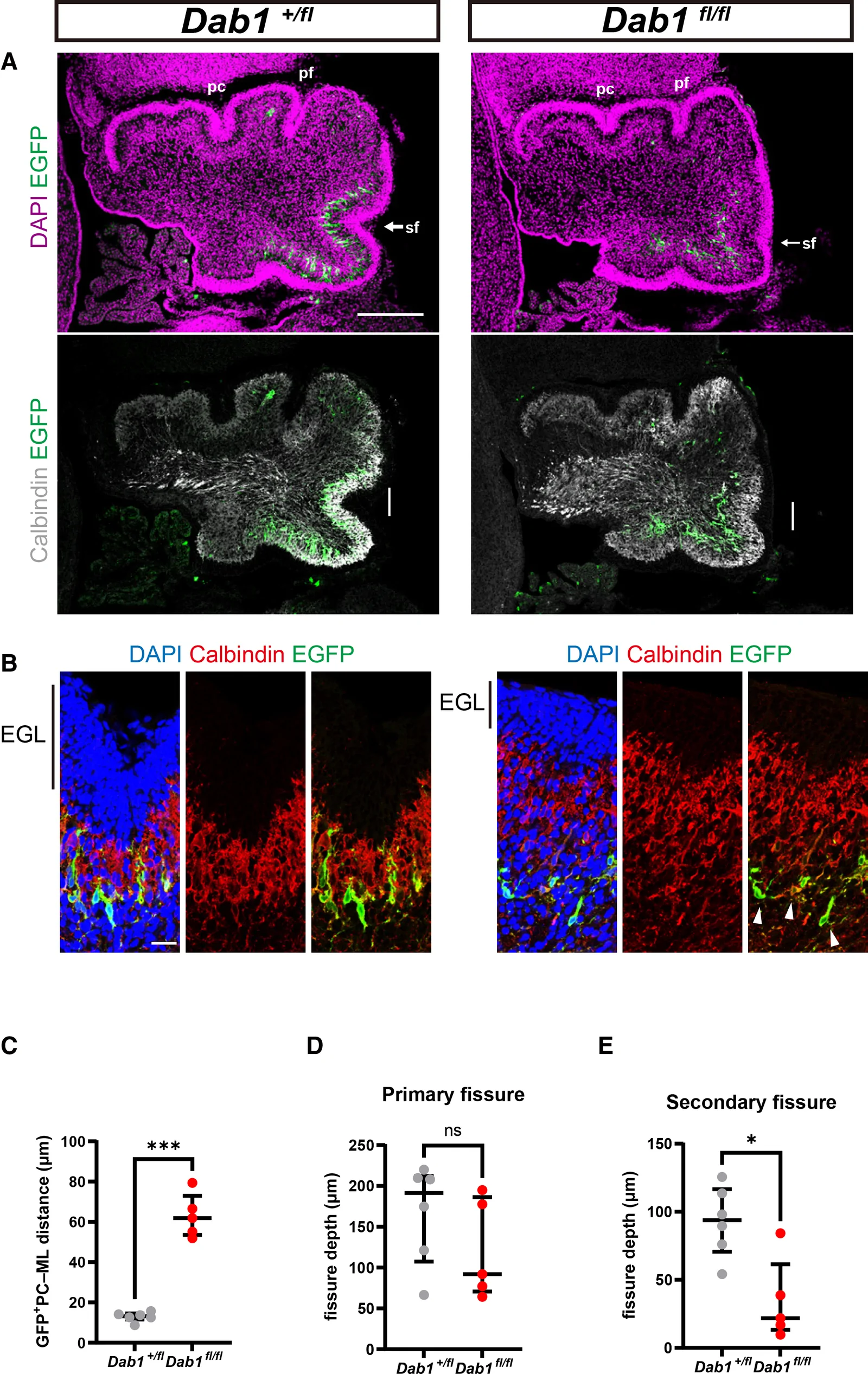
Embryonic *Dab1* deletion results in region-specific PC mispositioning and localized foliation defects (A) Sagittal cerebellar sections immunostained for EGFP (green), and Calbindin (white) with DAPI (magenta). Secondary fissure (sf) is indicated by arrows. (B) Higher-magnification view of the region indicated by the vertical lines in (A) Calbindin/GFP image, showing DAPI (blue), EGFP (green), and Calbindin (red). Arrowheads indicate ectopic PCs that have not reached the presumptive PCL position. (C) Quantification of the distance between EGFP-positive PCs at the folial surface and the ML in *Dab1*^*+/fl*^ and *Dab1*^*fl/fl*^ mice. (D and E) Quantification of fissure depth of primary fissure (pf) and sf. *Dab1 flox* mice were injected with AAV-L7-EGFP-P2A-Cre virus at E15, and cerebella were analyzed at P0. Scale bars, 200 μm (A) and 20 μm (B). PC–ML distance data are shown as mean ± SD (C) and fissure depth data are shown as median with interquartile range (D and E). *n* = 6 (*Dab1*^*+/fl*^) and 5 (*Dab1*^*fl/fl*^) mice. Statistical significance was assessed using unpaired two-tailed t tests for PC–ML distance (C) and the Mann-Whitney U test for fissure depth (D and E). pc, preculminate fissure.

To test whether the proposed model—that the timing of PC arrival near the cortical surface (presumptive PCL position just above the IGL) primarily determines the timing of fissure formation—broadly applies to cerebellar foliation, including fissures that arise during embryonic stages, we attempted to selectively perturb PC migration during embryogenesis. To achieve this, we injected AAV-L7-EGFP-P2A-Cre into *Dab1 flox* embryos at E15 and examined cerebella at P0. EGFP expression was detected in a subset of clusters of PCs (Figure 7A), reflecting that the L7 promoter is active only in a subset of PC clusters during embryonic stages.^42^^,^^43^^,^^44^ We found that, along the midline of the vermis, a cluster of EGFP-positive PCs consistently appeared in a restricted region of the future central-posterior lobes containing the secondary fissure (Figure 7A). Focusing on this site, EGFP-positive (*Dab1*-deficient) PCs in *Dab1*^*fl/fl*^ mice failed to reach near the cortical surface, whereas neighboring EGFP-negative PCs in *Dab1* ^*fl/fl*^ and EGFP-positive PCs in *Dab1* ^*+/fl*^ mice migrated to the presumptive PCL position near the cortical surface (Figures 7A-7C), suggesting that *Dab1* is also required for embryonic PC migration. Notably, the secondary fissure, which had already begun to form in *Dab1*^*+/fl*^ mice, was markedly impaired in *Dab1* ^*fl/fl*^ mice, while the primary fissure, which lacked EGFP-positive PCs, formed normally in both genotypes (Figures 7D and 7E). Thus, the foliation in places where PC migration was delayed was selectively impaired. These results together support the proposed model that the timing of the PC arrival at the presumptive PCL position locally helps determine local foliation patterns.

## Discussion

In this study, we demonstrate that the timing of PC migration to the presumptive PCL position is the primary determinant of cerebellar foliation by locally delivering SHH to GCPs. Furthermore, we show that spatial differences in the timing at which PCs reach the presumptive PCL position provide a biological explanation for the long-standing mystery of the temporal sequence of cerebellar foliation. Importantly, our P1-stage *Dab1* cKO—performed at a developmental window when PC monolayer and multilayer regions coexist—revealed regional differences in the timing of PC arrival, thereby unmasking foliation defects specifically in regions where the monolayer has yet to form. This approach also revealed that DAB1 is only required for PC migration until the PCs arrive at the presumptive PCL position and is thereafter dispensable. Failure of PCs to reach the presumptive PCL position reduces the local SHH-dependent expansion of GCPs and selectively disrupts fissure formation. Restoring SHH rescues this defective foliation despite persistent PC mispositioning. Based on these findings, we propose the following model for cerebellar foliation: during embryonic stages around E17, PCs disperse toward the cortical surface and form the multilayer, where the uppermost PCs have already reached the presumptive PCL position and begin delivering SHH to the overlying GCPs in the EGL, thereby initiating major fissure formation. After birth, most lobules quickly complete monolayer formation, whereas lobules VI/VII contain PC clusters that arrive substantially later, reaching the PCL around P4. This temporal lag results in a shifted temporal peak of GCP proliferation compared to other lobules, giving rise to later foliation that occurs postnatally.

Although abnormal PC positioning and cerebellar foliation defects have long been known to coexist in classical mutants such as *reeler*, *yotari*, and *cdf* mice,^45^ the mechanistic link between these two phenotypes has remained untested. Here, we show that even modest deviations of PCs from the PCL—far less severe than those seen in *reeler* and *yotari*—limit the amount of SHH that reaches GCPs and cause localized foliation defects. These findings define the formation of a PC monolayer as the specific positional condition necessary for the effective delivery of SHH and, consequently, for cerebellar foliation.

Several mechanisms may explain how the arrival of PCs at the presumptive PCL position increases the amount of SHH that reaches GCPs. The most straightforward explanation is a reduction in the distance between PCs and GCPs. When PCs reach the presumptive PCL, they are positioned closer to the EGL, potentially facilitating SHH delivery. In contrast, in lobules VI/VII of *Dab1* cKO mice, this distance remains greater. However, in this model, PCs are displaced by only a short distance (< 50 μm) from the presumptive PCL, suggesting that distance alone is unlikely to fully account for the observed reduction in SHH-dependent GCP proliferation. Therefore, additional mechanisms triggered by PC arrival at the presumptive PCL may also contribute.

PC dendrites extending toward the EGL may further facilitate SHH delivery. When PCs reach the presumptive PCL position, they undergo dendritic maturation (Figures 1K and 1Q), consistent with previous observations,^28^ and SHH immunoreactivity extends throughout the dendritic arbor (Figure 5B), as previously reported.^46^ Given that SHH is a lipid-modified ligand that remains membrane-tethered until released,^47^^,^^48^ the marked increase in dendritic membrane area is expected to enlarge the total surface from which SHH can be delivered to the overlying EGL. In addition, as PC dendrites extend, SHH released from these dendrites is delivered from sites in close proximity to GCPs, with minimal extracellular distance to traverse. In contrast, when PCs fail to reach the PCL position in *Dab1* cKO mice, SHH must traverse the IGL and ML to reach the EGL. During this process, SHH may be subject to diffusion constraints or degradation.

The idea that dendritic maturation of PCs enhances SHH delivery is consistent with the phenotype observed in *Bdnf* KO mice, in which impaired PC maturation coexists with foliation defects.^49^ In this model, the spf is absent and the other major fissures, which begin to form during the embryonic stages before dendrites are present, become shallower. This suggests that PC dendritic maturation enhances the depth of foliation in these major fissures, and that PC dendritic maturation-mediated GCP proliferation is a general mechanism for foliation across the cerebellum.

We further refined the role of DAB1 during the cerebellar development. Although the analysis of *yotari* mutants has shown that DAB1 is needed at least for the initial step of PC dispersion, it remains unclear how far along PC migration DAB1 is required. Our findings sharpen this understanding by demonstrating that DAB1 is necessary only until PCs reach the presumptive PCL position and thereafter dispensable. In addition, we show that DAB1’s role in foliation is mediated through establishing correct PC positioning, not by directing PC-intrinsic programs. These insights refine the mechanistic interpretation of the cerebellar foliation defects in *yotari* and *reeler* mice^50^—conditions that mirror Reelin-related cerebellar foliation defects in humans^51^—by indicating that failed PC migration is the primary defect and the resulting reduction in SHH delivery to GCPs due to the markedly increased PC-GCP distance impairs foliation as a secondary consequence.

Our findings demonstrate that DAB1 is required for the transition from a multilayer to a monolayer organization of PCs. Although it remains controversial whether PCs use BG fibers as a scaffold for migration^52^^,^^53^^,^^54^^,^^55^ (reviewed by Schilling^56^), static histological analyses have suggested that PCs migrate along BG fibers extending toward the pial surface.^2^ Furthermore, the extracellular matrix molecules, including Tenascin-C, are present around PC during this period.^57^ Given the known function of Reelin signaling in controlling the interactions between neurons and the extracellular environment, including glial scaffold and extracellular matrix, during neuronal migration, interactions between PCs and extracellular factors may be regulated by the Reelin signal. Alternatively, Reelin signal might control PC migration in a cell-autonomous manner. Indeed, during the early phase of PC migration, a Reelin-dependent switchback-like movement of PCs in the lateral-dorsal cerebellum was reported, in which PCs appear to cross BG fibers.^58^ Since control of the cytoskeleton is crucial for PC migration^59^ and the Reelin signal regulates neuronal migration autonomously via cytoskeletal control in several contexts,^60^^,^^61^^,^^62^ Reelin-mediated control of cytoskeleton may contribute to PC migration. Future studies are needed to clarify how PCs interpret Reelin signaling to reach the presumptive PCL position.

Finally, our data provide a mechanistic framework that helps explain how cerebellar fissures emerge sequentially over time. Previous work has suggested that lobules VI/VII follow a developmentally delayed program. *Gli1* expression in lobules VI/VII is delayed relative to other lobules.^9^ Legue et al. demonstrated that the temporal peak of GCP production and the EGL thickening occur later in lobules VI/VII and speculated that these delays might underlie the late formation of the spf.^15^ Our results suggest that the timing of PC arrival at the presumptive PCL position acts as a developmental timer, specifying when SHH-dependent GCP expansion initiates and thereby controlling the temporal sequence of fissure formation. By selectively perturbing PC positioning and restoring SHH signaling, our experiments provide direct mechanistic evidence linking the arrival timing of PCs to the temporal sequence of foliation.

It is important to note that our proposed axis of PC migration-SHH delivery-foliation does not imply that PCs instruct where a fissure will form. Although the EGL thickened inwardly at future fissures before foliation, PCs are not observed to arrive earlier at these sites,^38^ and we similarly did not detect any earlier PC arrival in our analyses. Furthermore, GCP proliferation at future fissures is not elevated relative to surrounding regions.^16^ Instead, in our model, PCs influence foliation through the collective dynamics of their migration. PCs migrate as clusters,^37^^,^^63^ and the monolayer formation observed later in lobules VI/VII likely reflects a delay in the arrival of clusters at the presumptive PCL position. Such a delay collectively postpones SHH delivery to GCPs, shifting the temporal peak of GCP expansion and thereby driving sequential foliation.

Although PCs in both lobules VI/VII and X complete monolayer formation later than in other lobules, only lobules VI/VII generate an additional fissure. Notably, lobule X shows a relatively thick EGL already by P2 (Figure 6A) and a less pronounced increase in GCP proliferation toward P6–P8 compared with lobules VI/VII. Because lobule X lies closer to the ventricular zone, PCs in this region travel a much shorter distance during dispersion.^37^ As a result, the difference in SHH accessibility before and after PC arrival at the presumptive PCL position may be less pronounced than in lobules VI/VII, which are the most distant from the ventricular zone. In lobules VI/VII, delayed PC migration is likely to create a more substantial temporal shift in SHH-dependent GCP expansion. In *Dab1* cKO mice, despite abnormal PC positioning in lobule X, we did not observe a clear effect on the parafloccular fissure (data not shown). This may be because, as discussed above, the impact on GCP proliferation is relatively mild in lobule X, and because PCs in the adjacent lobule IX, which are likely to contribute to parafloccular fissure formation, can reach the PCL position.

Although a delayed peak of GCP expansion appears necessary for generating a late-forming fissure, it is unlikely to be sufficient on its own. Additional factors likely influence whether a shifted proliferative wave ultimately produces a fold. Lawton et al. proposed that anchoring centers (ACs)—distinct cytoarchitectures identified by Sudarov and Joyner at the base of each fissure^38^—establish mechanically stable boundaries that subdivide the cerebellum into self-similar domains capable of further folding.^16^ Within its framework, the distinctive morphology of lobules VI/VII may provide a permissive environment in which delayed GCP expansion could contribute to the formation of the spf.

In summary, our study establishes that PC migration to the presumptive PCL position is a key developmental event that enables effective SHH delivery to GCPs and thereby drives cerebellar foliation. Spatial differences in the timing of PC arrival at the presumptive PCL position primarily control the temporal sequence of fissure formation, providing the mechanistic explanation for this fundamental developmental phenomenon.

### Limitations of the study

Our analysis primarily focuses on the mouse cerebellar vermis; therefore, it remains unclear whether the PC migration-SHH delivery-foliation axis we propose extends to other fissures in the mouse cerebellar hemispheres and the human cerebellum. Future work will be required to test the generality of this mechanism across cerebellar regions and species.

In the present study, our analysis of *Dab1* cKO mice was limited to P15, and we did not assess whether the foliation defects and abnormal PC positioning persist in adulthood. In addition, given that hypoplasia of lobules VI/VII has been associated with neurodevelopmental disorders, including autism spectrum disorder,^64^^,^^65^ the long-term consequences of the observed foliation defects remain of considerable interest. Future studies should evaluate both motor and non-motor functions associated with these structural abnormalities.

## Resource availability

### Lead contact

Further information and requests for resources and reagents should be directed and will be fulfilled by the lead contact, Kazunori Nakajima (kazunori@keio.jp).

### Materials availability

All materials generated in this study are available upon request to the lead contact.

### Data and code availability

• Data reported in this paper will be shared by the lead contact upon request.
• This study did not generate large-scale datasets or custom code.
• Any additional information required to reanalyze the data reported in this paper is available from the lead contact upon request.

## Acknowledgments

We thank Dr. Hideaki Imai (Kobe University, Kobe, Japan) and Dr. Yu Katsuyama (Shiga Medical University, Otsu, Japan) for providing *Dab1 flox* mice, and Dr. Mitsuharu Hattori (Nagoya City University) for *reeler* mice on the ICR background. We also thank Karel Svoboda (Howard Hughes Medical Institute) for providing pAAV-CAG-EGFP, the University of Pennsylvania Penn Vector Core for pAdΔF6 and pAAV2/9, and Dr. Hirokazu Hirai (Gunma University) for pAAV-L7-6-EGFP. This work was supported by JST, 10.13039/501100025019SPRING program to R.U.; 10.13039/501100001691JSPS 10.13039/501100001691KAKENHI grant nos. JP20K06670, JP23H02491, and JP24H01972 to Y.H.; JP24K09668 and JP21K06413 to T.H.; JP20H05688 and JP25K02437 to K.N.; 10.13039/100007684Asahi Glass Foundation to Y.H.; 10.13039/100007449Takeda Science Foundation to Y.H. and K.N.; Keio Gijuku Fukuzawa Memorial Fund for the Advancement of Education and Research to K.N.; and Keio Gijuku Academic Development Funds to Y.H. and K.N.; AMED under grant no. JP25gm6910028 to Y.H.

## Author contributions

Conceptualization, R.U., Y.H., and K.N.; methodology, R.U., T.H., Y.H., and K.N.; investigation, R.U., T.H., T.K., Y.H., and K.N.; visualization, R.U.; supervision, Y.H. and K.N.; writing – original draft, R.U.; writing – review and editing, Y.H. and K.N. R.U. performed most of the experiments. T.H. and T.K. performed the initial analyses of *Dab1* cKO mice using the AAV-CAG-EGFP-P2A-Cre virus (Figure S1).

## Declaration of interests

The authors declare no competing interests.

## Declaration of generative AI and AI-assisted technologies in the writing process

During the preparation of this manuscript, the authors used ChatGPT (OpenAI) to assist with language refinement. All content was reviewed and edited by the authors, who take full responsibility for the final publication.

## STAR★Methods

### Key resources table

**Table:** 

REAGENT or RESOURCE	SOURCE	IDENTIFIER
**Antibodies**		

Rabbit anti GFP	MBL	Cat#: 598; RRID: AB_591819
Chicken anti GFP	abcam	Cat#: ab13970; RRID: AB_300798
Goat anti Calbindin	Nittobo	Cat#: MFSR100400; RRID: AB_2571569
Rabbit anti BLBP	Millipore	Cat#: ABN14; RRID: AB_10000325
Chicken anti Nestin	Aves lab	Cat#: NES; RRID: AB_2314882
Rabbit anti Ki67	Thermo Fisher	Cat#: FM9-106-S1; RRID: AB_2341197
Mouse anti BrdU	BD biosciences	Cat#: 347580; RRID: AB_400326
Rabbit anti SHH	Santa Cruz	Cat#: sc9024; RRID: AB_2239216
Rabbit anti RORα	Santa Cruz	Cat#: sc28612; RRID: AB_28612
Mouse anti SHH	Santa Cruz	Cat#: sc365112; RRID: AB_10709580
Rabbit anti Calbindin	Nittobo	Cat#: MFSR100390; RRID: AB_2571568
Alexa Fluor 488 donkey anti-rabbit IgG	Invitrogen	Cat#: A21206; RRID: AB_2535792
Alexa Fluor 488 donkey anti-chicken IgY	Jackson Immuno Research	Cat#: 703-545-155; RRID: AB_2340375
Alexa Fluor 555 donkey anti-goat IgG	Invitrogen	Cat#: A21432; RRID: AB_141788
Alexa Fluor 555 donkey anti-rabbit IgG	Invitrogen	Cat#: A31572; RRID: AB_162543
Alexa Fluor 555 donkey anti-mouse IgG	Invitrogen	Cat#: A31570; RRID: AB_2536180
Alexa Fluor 594 donkey anti-chicken IgY	Jackson Immuno Research	Cat#: 703-585-155; RRID: AB_2340377
Alexa Fluor 647 donkey anti-goat IgG	Invitrogen	Cat#: A21447; RRID: AB_2535864
HRP-conjugated goat anti-mouse IgG	Jackson Immuno Research	Cat#: 115-035-003; RRID: AB_10015289
HRP-conjugated goat anti-rabbit IgG	Jackson Immuno Research	Cat#: 111-035-003; RRID: AB_2313567

**Bacterial and virus strains**		

AAV-CAG-EGFP-P2A-Cre	This paper	N/A
AAV-L7-EGFP-P2A-Cre	This paper	N/A
AAV-CAG-DIO-SHH	This paper	N/A

**Chemicals, peptides, and recombinant proteins**		

AAV-MAX Helper-Free AAV Production System	Thermo Fisher Scientific	Cat#: A49784
Benzonase nuclease	Millipore	Cat#: 70746
POROS CaptureSelect AAV Affinity Resin	Thermo Fisher Scientific	Cat#: A36739
DNaseI	SIGMA	Cat#: DN25
Bromodeoxyuridine (BrdU)	SIGMA	Cat#: B9285
ISHpalette Short hairpin amplifier, Cyanine5-S72	Nepagene	Cat#: IPL-B-C-S72
protease inhibitor	Roche	Cat#: 11697498001
PhosSTOP	Roche	Cat#: 04906837001
Can Get signal 1 solution	TOYOBO	Cat#: NKB-101
Chemilumi One Super	Nacalai Tesque	Cat#: 02230-30

**Experimental models: Organisms/strains**		

*Dab1 flox* mouse	Gift from Hideaki Imai and Yu Katsuyama (Imai et al.^66^)	N/A
C57BL/6N mouse	Japan SLC	RRID: IMSR_JAX:000664
*reeler* mouse (B6CFe *a/a-Reln*^*rl*^/J)	The Jackson Laboratory	RRID: IMSR_JAX:000235
*reeler* mouse (ICR)	Gift from Mitsuharu Hattori	N/A

**Recombinant DNA**		

pAAV-CAG-EGFP	Gift from Karel Svoboda	RRID: Addgene_28014
pAAV-CAG-EGFP-P2A-Cre	This paper	N/A
pAAV-L7-6-EGFP	Gift from Hirokazu Hirai (Nitta et al.^67^)	N/A
pAAV-L7-EGFP-P2A-Cre	This paper	N/A
pAAV-CAG-DIO	Honda et al.^68^	N/A
pAAV-CAG-DIO-SHH	This paper	N/A

**Software and algorithms**		

Prism 11 for Windows	Graphpad	https://www.graphpad.com
ImageJ	NIH	https://imagej.net

### Experimental model and study participant details

#### Mice

*Dab1 flox* mice^66^ were kindly provided by Dr. Hideaki Imai (Kobe University, Kobe, Japan) and Dr. Yu Katsuyama (Shiga Medical University, Otsu, Japan). They were maintained on a C57BL/6 background, and littermate *Dab1*^*+/fl*^ mice served as controls for all *Dab1* cKO experiments. Because cerebellar foliation differs across strains,^13^ and to match the genetic background of the *Dab1 flox* line, C57BL/6N mice (purchased from Japan SLC) were used as wild type (Figure 6). The *reeler* (*rl*^−/−^) mice (B6CFe *a/a-Reln*^*rl*^/J; RRID: IMSR_JAX:000235) were originally obtained from The Jackson Laboratory and have been maintained in our facility. For embryonic analyses (Figures 5F–5I; Figure S3), we additionally used a *reeler* line that had been backcrossed onto the ICR background for at least 10 generations, which was kindly provided by Dr. Mitsuharu Hattori (Nagoya City University).

All mice were housed in a specific pathogen-free (SPF) facility and maintained on a 12-h light/dark cycle (lights on at 8:30 a.m.) at 24 °C. All animal procedures were approved by the Institutional Animal Care and Use Committees of Keio University (A2021-030) and were conducted in accordance with institutional guidelines at Keio University and the Japanese government regulations for animal experimentation. Noon on the day the vaginal plug was found was designated as embryonic day (E)0.5. Mice of both sexes were used in this study.

### Method details

#### Plasmids

pAAV-CAG-EGFP-P2A-Cre was generated from the pAAV-CAG-EGFP backbone (a gift from Karel Svoboda, Addgene plasmid #28014; http://n2t.net/addgene:28014 RRID: Addgene_28014)^69^ by replacing the original EGFP cassette with an expression cassette assembled by sequential insertion of EGFP, a P2A peptide linker, and Cre recombinase into a modified multiple cloning site created by BamHI/HindIII excision. pAAV-L7-EGFP-P2A-Cre was generated from pAAV-L7-6-EGFP (a gift from Dr. Hirokazu Hirai, Gunma University).^67^ The bGH polyadenylation signal was replaced with the SV40 polyadenylation signal, and a P2A-Cre cassette (derived from pAAV-CAG-EGFP-P2A-Cre) was inserted downstream of the L7-6 promoter. To generate pAAV-CAG-DIO-SHH, total RNA was extracted from E18.5 C57BL/6JJcl mouse brain and reverse-transcribed into cDNA. The coding sequence of mouse Shh was then amplified by PCR using the following primers: 5′-gccaccatgctgctgctgctggccag-3′ and 5′-tcagctggacttgaccgccattc-3' (Kozak sequence is underlined). The obtained product was cloned into the pAAV-CAG-DIO1 vector.^68^ All cloning junctions and open reading frames were confirmed by Sanger sequencing. The adenovirus helper plasmid pADΔF6 and vector pAAV2/9 were kindly provided by the University of Pennsylvania Penn Vector Core.

#### AAV production and purification

Recombinant AAV (rAAV) was produced and purified using two protocols. For experiments performed earlier in the study, rAAV production and purification were carried out as described previously.^68^ Briefly, transfected HEK293T cells were harvested and lysed by repeated freeze–thaw cycles, and viral particles were purified by iodixanol gradient ultracentrifugation. In subsequent experiments, rAAV was produced using the AAV-MAX Helper-Free AAV Production System (Thermo Fisher Scientific; Cat# A49784) according to the manufacturer’s instructions. HEK293-derived Viral Production Cells 2.0 were cultured in Viral Production Medium and transfected with pADΔF6 and vector pAAV2/9 together with the transfer plasmids: pAAV-CAG-EGFP-P2A-Cre, pAAV-L7-EGFP-P2A-Cre, or pAAV-CAG-DIO-SHH. At 72 h after transfection, cells and medium were harvested and lysed with AAV-MAX Lysis Buffer supplemented with Benzonase nuclease (Millipore, Cat# 70746), followed by clarification and affinity purification using POROS CaptureSelect AAV Affinity Resin (ThermoFisher Scientific; Cat# A36739). In both protocols, eluted rAAV was finally buffer-exchanged into sterile PBS and concentrated using Vivaspin centrifugal concentrators, 100 kDa MWCO (Sartorius; Cat# VS2042).

#### Viral genome titer quantification

For determination of genomic titers, purified rAAV samples were treated sequentially with DNaseI (SIGMA, Cat# DN25) to remove unpackaged DNA and Proteinase K to release encapsidated viral genomes. Viral genomes were quantified by qPCR using a StepOnePlus Real-Time PCR System (Applied Biosystems) with a TaqMan probe-based assay targeting the WPRE sequence. Standard curves were generated using linearized plasmid DNA. Primer and probe sequences are listed in Table S1.

#### AAV injection

For postnatal injections, pups were anesthetized on ice and 1.5 μL of rAAV mixed with 0.01% Fast Green was delivered into each lateral ventricle bilaterally. For embryonic injections, pregnant females were anesthetized by intraperitoneal administration of an anesthetic mixture of medetomidine hydrochloride, midazolam, and butorphanol, and the uterine horns were gently exteriorized. rAAV was then injected unilaterally into the lateral ventricle of E15.0 embryos. All injections were performed using a Narishige IM-300 microinjector with pulled glass capillaries (Narishige, GD-1). Injection volumes and viral genomic titers for each experiment are summarized in Table S2.

#### BrdU labeling

Bromodeoxyuridine (BrdU; SIGMA, Cat# B9285) was dissolved in sterile PBS at 5-10 mg/mL and administered to pups by subcutaneous injection at 0.1 mg/g body weight (equivalent to 100 mg/kg). Cerebella were collected 1 h after BrdU administration.

#### Tissue preparation and sectioning

Mice were transcardially perfused with phosphate-buffered saline (PBS) followed by 4% paraformaldehyde (PFA) in 0.1M phosphate buffer (pH7.4). Brains were then isolated and postfixed in 4% PFA on ice (2 h for P5–P15; 1 h for P0–P4). Stereomicroscopic images of the cerebellum were acquired from representative samples after applying 0.03% Fast Green dissolved in PBS to enhance the contrast of cerebellar fissures. After washing in PBS, tissues were cryoprotected in 25% sucrose/PBS overnight, embedded in a mixture of optimal cutting temperature (OCT) compound (Sakura Finetek) and 30% sucrose/PBS, and frozen in liquid nitrogen. Cryosections were cut sagittally at 20 μm using a Leica CM3050 S cryostat.

#### Immunohistochemistry

Cryosections were brought to room temperature, rinsed in PBS, and, when required, subjected to antigen retrieval (citrate buffer, 105 °C for 10 min). Sections were then incubated in blocking solution (5% donkey serum and 0.1% Triton X-100 in PBS; 0.3% Triton X-100 was used for anti-SHH staining) for 30 min at room temperature. Primary antibodies were diluted in the corresponding blocking solution and applied overnight at 4 °C. After PBS washes, sections were incubated with species-specific fluorescent secondary antibodies for 2 h at room temperature, counterstained with DAPI, and mounted with PermaFluor (Thermo Fisher Scientific). The primary and secondary antibodies used in this study are listed in Table S3.

#### Fluorescent *in situ* hybridization

Hybridization chain reaction (HCR)-based fluorescent *in situ* hybridization was performed using the ISH palette HCR kit (Nepagene, Japan) according to the manufacturer’s protocol. Split probes, which were concatenated with initiator sequences for HCR amplification, were designed against coding sequences of *Shh* and *Gli1* (Table S4). Cryosections (20 μm) were brought to room temperature, rinsed in PBS, dehydrated in 100% methanol for 10 min, and rehydrated in PBS containing 0.1% Tween 20 (PBST). After incubation in the hybridization buffer (Nepagene) for 5 min, split-probe sets targeting *Shh* or *Gli1* were diluted in hybridization buffer and hybridized overnight at 37°C. After hybridization, sections were washed in 0.5× SSC containing 0.1% Tween 20 (SSCT) at 37 °C (3 × 10 min each). Signal amplification was performed by incubating sections with ISH short hairpin amplifier (IPL-B-C-S72) together with DAPI for 2 h at room temperature. Sections were finally washed in PBST and mounted in PermaFluor (Thermo Fisher Scientific).

#### Western blot analysis for cerebellar lysates

Cerebella were rapidly dissected in ice-cold PBS and homogenized in tissue lysis buffer [50 mM Tris-HCl (pH 7.5), 150 mM NaCl, 10% glycerol, 1% Triton X-100, 50 mM NaF, and 1 mM Na_3_VO_4_] supplemented with protease inhibitor (Roche, Cat# 11697498001) and PhosSTOP (Roche, Cat#04906837001), using a pellet mixer. Samples were further lysed using a sonicator (Bioruptor UCD-200TM, Cosmo Bio; 15 s on/15 s off, 10 min). Extracts were cleared by centrifugation at 15,000 rpm for 15 min at 4°C. Protein samples were mixed with 4× Laemmli sample buffer, boiled at 95°C for 5 min, and subjected to SDS-PAGE on 4-20% polyacrylamide gels. Proteins were transferred onto polyvinylidene difluoride (PVDF) membranes. Membranes were blocked in 5% BSA in PBST (0.05% Tween 20 in PBS) for 30min and incubated with primary antibodies diluted in Can Get signal 1 solution (TOYOBO, #Cat NKB-101) overnight at 4°C on a rocking platform. After washing with PBST, membranes were incubated with horseradish peroxidase-conjugated secondary antibodies in PBST for 1 h at room temperature on a rocking platform. Signals were detected using Chemilumi One Super (Nacalai Tesque, #Cat 02230-30) and captured with a cooled CCD camera system (LAS-4000 mini; GE Healthcare). Relative band intensities were quantified by measuring the integrated density of each band, subtracting the background from the same lane, and normalizing to the background-subtracted integrated density of Calbindin, which serves as a PC-specific loading reference. The following primary antibodies were used: anti-SHH (mouse; Santa Cruz, sc-365112; 1:100) and anti-Calbindin (rabbit; Nittobo, MFSR100390; 1:5,000). HRP-conjugated secondary antibodies were obtained from Jackson ImmunoResearch (anti-mouse, 115-035-003; anti-rabbit, 111-035-003; 1:1,000).

### Quantification and statistical analysis

#### Image quantification

Image quantification was performed using ImageJ software (NIH). Fissure depth measurements of the primary fissure and secondary fissure were performed on midline sagittal sections of the cerebellar vermis, defined as sections in which no cerebellar nuclei were visible. Fissure depth was measured as the distance from the fissure base to the pial surface. For spf measurements, four sagittal sections of the cerebellar vermis spaced at 160 μm intervals were analyzed for each cerebellum, and the maximal spf depth was used for quantification. These sections spanned the vermis from the midline to just before the level at which continuity between the cerebellar white matter and the pons became visible. The distance between EGFP-positive PCs and the ML was quantified by measuring, for each cell, the distance from the center of the PC soma to the lower boundary of the ML in the superficial region of each lobule. For quantification of EGL thickness, midline sagittal sections of the cerebellar vermis were cropped to a 150-μm-wide region along the EGL. EGL thickness was measured at three predefined, evenly spaced positions within this region, and the mean value was used for analysis. For BrdU analysis, midline sagittal sections of the cerebellar vermis were cropped to the outer 150 μm of the EGL. BrdU-positive and DAPI-positive cells within this region were counted. BrdU quantification was performed in a blinded manner after image cropping. Fluorescence intensity of SHH and Calbindin was quantified in individual PCs. For IHC quantification of SHH normalized to Calbindin, mean intensity was measured within the soma while excluding the DAPI-positive nuclear region. Complete blinding was not feasible for *Dab1* cKO samples because of overt PC mispositioning; therefore, all measurements were conducted using predefined, objective criteria.

#### Statistical analysis

All statistical analyses were performed using GraphPad Prism (GraphPad Software). Data are generally presented as mean ± SD and statistical significance was assessed using two-tailed Welch’s t-tests. For comparisons involving multiple factors or groups, two-way ANOVA with Sidák’s multiple comparisons test was used for analyses of PC–ML distance across lobules (Figures 1J and 1R; Figure S1G) and one-way ANOVA with Tukey’s multiple comparisons test was used for analyses of EGL thickness in *reeler* mice (Figures 3F and 3G). For datasets showing large variance, fissure depth measurements in Figures 2I, 7D, and 7E were analyzed using the Mann–Whitney U test and are presented as median with interquartile range. For all experimental analyses, each *n* represents an individual mouse. A *p* value less than 0.05 was considered significant. All significant statistical results are indicated within the figures with the following conventions: ∗*p* < 0.05, ∗∗*p* < 0.01, ∗∗∗*p* < 0.001, ∗∗∗∗*p* < 0.0001. Exact *n* values and statistical tests used are indicated in the figure legends. Exact *p* values, test statistics (t, U, and F values), and degrees of freedom (for t-tests and ANOVAs) are provided in Table S5.
