# Supplementary material for: Purkinje cell arrival at the Purkinje cell layer orchestrates cerebellar foliation and its temporal sequence
Source: iScience. 2026 Sep 1;29(9):116664. doi: 10.1016/j.isci.2026.116664 (PMC13562430; doi:10.1016/j.isci.2026.116664)
Supplement: Supplementary file 1 [file mmc1.pdf]

iScience, Volume *29*

Supplemental information

Purkinje cell arrival at the Purkinje cell layer orchestrates cerebellar foliation and its temporal sequence

Ryosuke Uchi, Takao Honda, Takuto Kasai, Yuki Hirota, and Kazunori Nakajima

Figure S1

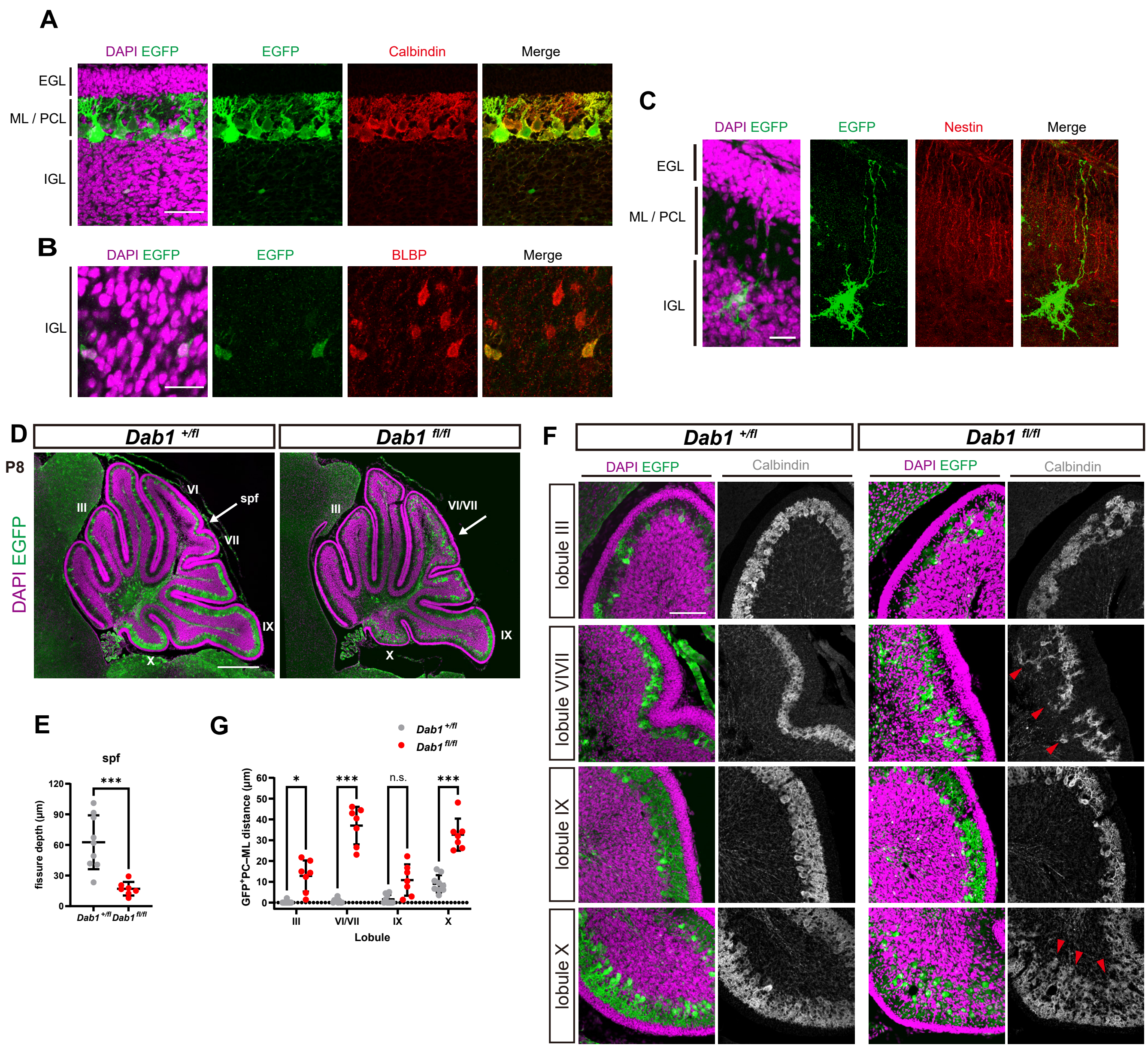
A
DAPI EGFP
EGFP
Calbindin
Merge
EGL
ML / PCL
IGL
B
DAPI EGFP
EGFP
BLBP
Merge
IGL
C
DAPI EGFP
EGFP
Nestin
Merge
EGL
ML / PCL
IGL
D
Dab1 +/fl
Dab1 fl/fl
P8
DAPI EGFP
III
VI
spf
VII
IX
X
VI/VII
F
Dab1 +/fl
Dab1 fl/fl
DAPI EGFP
Calbindin
lobule III
lobule VI/VII
lobule IX
lobule X
E
spf
fissure depth (μm)
Dab1 +/fl
Dab1 fl/fl
G
GFP+PC–ML distance (μm)
Lobule
n.s.

Figure S1. Postnatal Dab1 deletion driven by a CAG promoter preferentially disrupts PC layer organization in lobules VI/VII and X, with impaired spf formation, related to Figure 1.

(A–G) *Dab1 flox* mice were injected with AAV-CAG-EGFP-P2A-Cre virus at P1, and cerebella were analyzed at P8. (**A–C**) Sagittal cerebellar sections from *Dab1 +/fl* mice immunostained for EGFP (green) together with Calbindin (A, red), BLBP (B, red), or Nestin (C, red), with DAPI (magenta). (**D**) Sagittal sections of the cerebellar vermis. Superior posterior fissure (spf) is indicated by an arrow. (**E**) Quantification of the spf depth. (**F**) Higher-magnification images of lobule III, VI/VII, IX and X, showing PC layer disruption in lobule VI/VII and X in *Dab1 fl/fl* mice (red arrowheads). (**G**) Quantification of the distance between EGFP-positive PCs at the folial surface and the molecular layer (ML). Scale bars, 50 µm (A), 20 µm (B, C), and 500 µm (D), 200 µm (F). Data are shown as mean ± SD. *n* = 7-9 mice per genotype. Statistical significance was assessed using unpaired two-tailed *t* tests. EGFP-positive PC–ML distance across lobules was analyzed using two-way ANOVA with Sidák's multiple comparisons test. EGL, external granule layer; PCL, Purkinje cell layer; ML, molecular layer; IGL, internal granular layer.

Figure S2

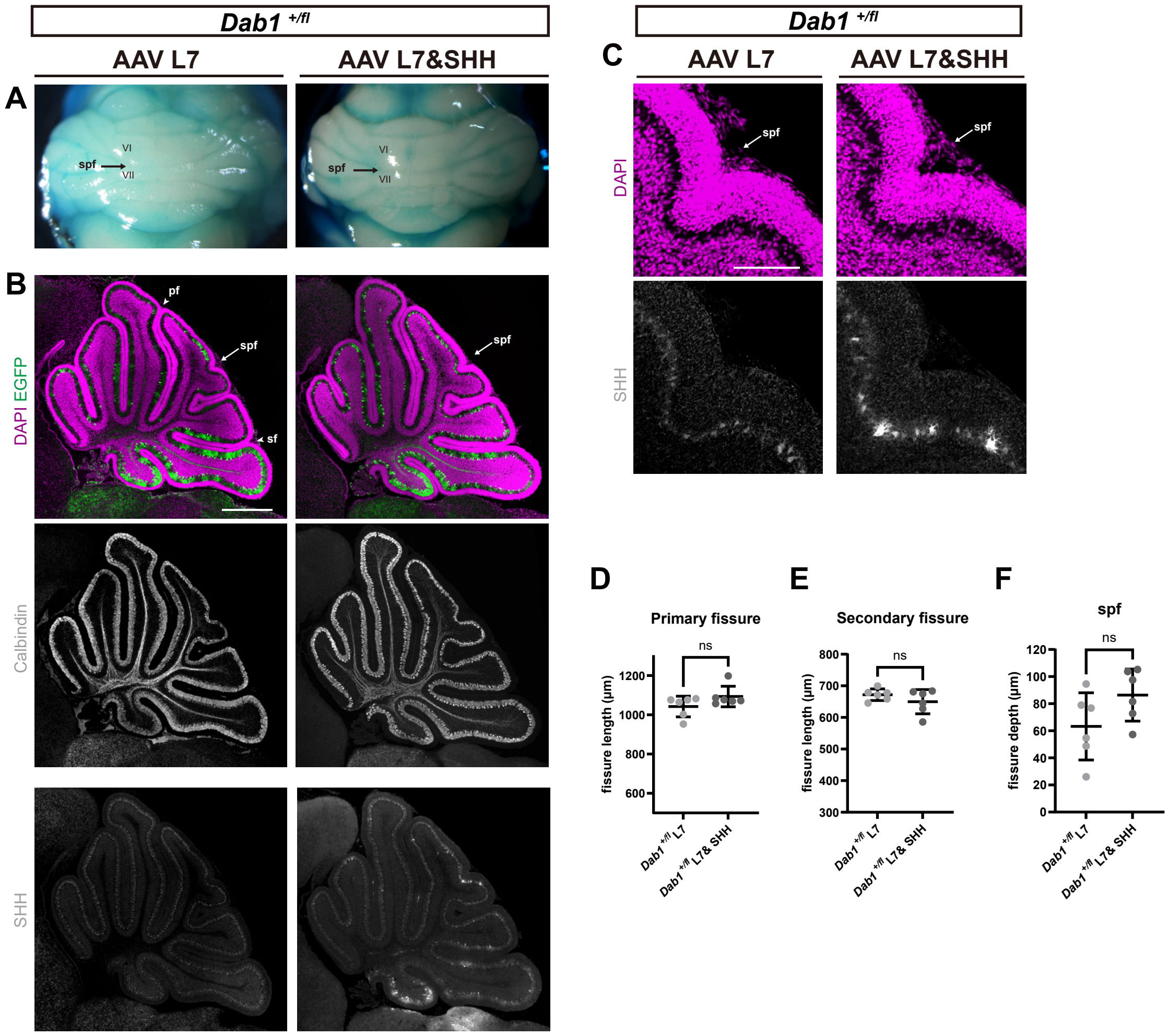

Dab1 +/fl
AAV L7
AAV L7&SHH
A
spf
VI
VII
B
DAPI EGFP
pf
spf
sf
Calbindin
SHH
C
DAPI
SHH
D
Primary fissure
ns
fissure length (μm)
1200
1000
800
600
Dab1 +/fl L7
Dab1 +/fl L7& SHH
E
Secondary fissure
ns
800
700
600
500
400
300
F
spf
ns
fissure depth (μm)
120
100
80
60
40
20
0

Figure S2. AAV-mediated SHH expression does not alter cerebellar foliation in *Dab1 +/fl* mice, related to Figure 4.

(A–F) *Dab1 +/fl* mice were injected with AAV at P1 with AAV-L7-EGFP-P2A-Cre alone or with AAV-L7-EGFP-P2A-Cre together with AAV-CAG-DIO-SHH, and the cerebella were analyzed at P8. (**A**) Stereomicroscopic images of the cerebellum. The superior posterior fissure (spf) is indicated by arrows. (**B**) Sagittal cerebellar sections immunostained for EGFP (green), Calbindin (white), and SHH (white) with DAPI (magenta). (**C**) Higher-magnification view of lobules VI/VII. The spf is indicated by arrows. (**D–F**) Quantification of the depth of primary fissure (pf) (D), secondary fissure (sf) (E), and spf (F). Scale bars, 500 µm (B) and 100 µm (C). Data are shown as mean ± SD. $n = 6$ mice per group. Statistical significance was assessed unpaired two-tailed t tests.

Figure S3

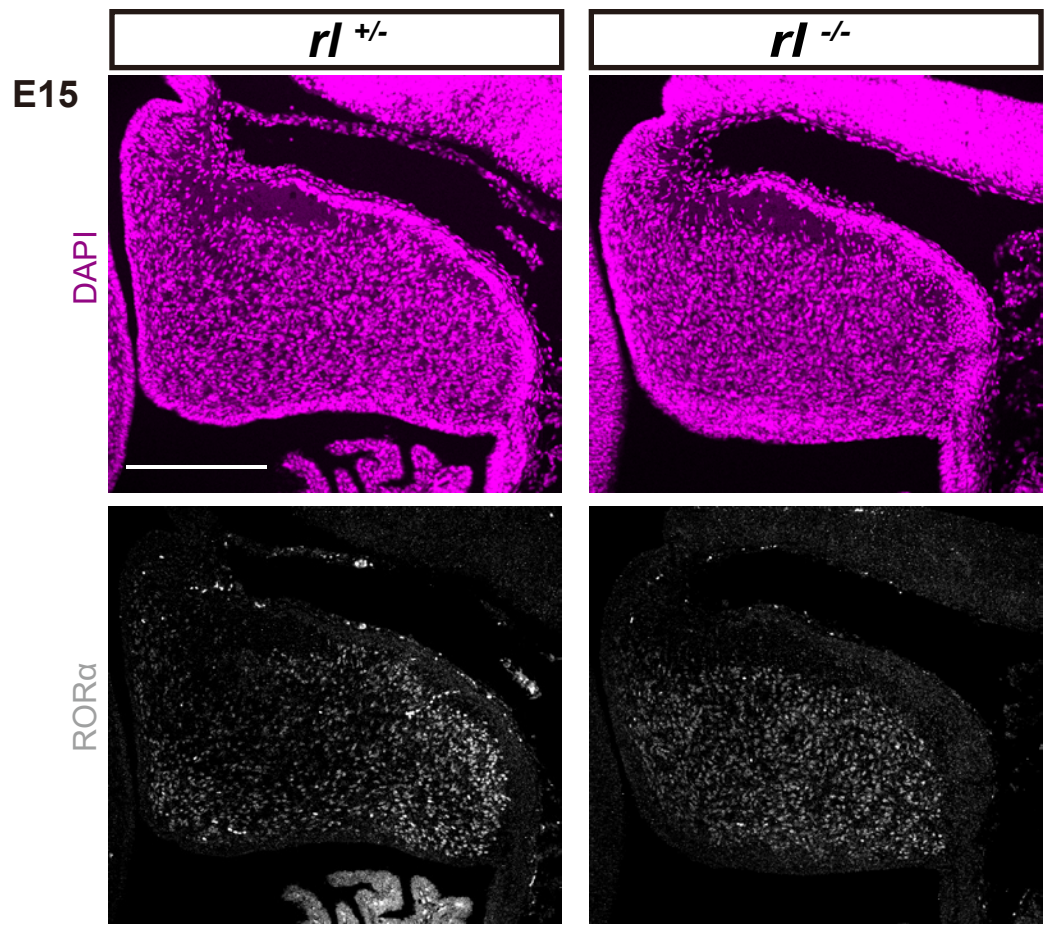

Figure S3. PC positioning in *reeler* mice at E15, related to Figure 5.

Sagittal cerebellar sections from *reeler* mice at E15, immunostained for RORα (white) with DAPI (magenta). Scale bars, 200 μm.

Table S1. qPCR primers and probe used in viral genome titer quantification, related to STAR methods.

**Forward primer**	5′-GGGCCACAACTCCTCA TAAAA-3′
**Reverse primer**	5′-ATACGCGTGC TTTAAGTGCCTTTG-3′
**Probe**	5′-/56-FAM/TCATGCTAT/ZEN/TGCTTCCCGTATGGCT/3IABkFQ/-3′

Table S2. AAV injection parameters and genomic titers, related to STAR methods.

Figure	Vector	Titer (vg/mL)	Injection volume	Injection stage
Figure S1	CAG-EGFP-P2A-Cre	5.0 × 10^12	1.5μL (bilateral)	P1
Figure 1	L7-EGFP-P2A-Cre	1.4 × 10^13	1.5μL (bilateral)	P1
Figure 2	L7-EGFP-P2A-Cre	1.4 × 10^13	1.5μL (bilateral)	P4
Figure 3	CAG-EGFP-P2A-Cre	4.0 × 10^11	1.5μL (bilateral)	P1
Figure 4	L7-EGFP-P2A-Cre	7.0 × 10^12	1.5μL (bilateral)	P1
	CAG-DIO-SHH	1.2 × 10^12	1.5μL (bilateral)	
Figure 7	L7-EGFP-P2A-Cre	1.4 × 10^13	0.5-1μL (unilateral)	E15

Table S3. Antibodies used for IHC, related to STAR methods.

A. Primary antibody

Antibody	Host	Source	Catalog Number	Antigen retrieval	Concentration used
GFP	Rabbit	MBL	598	Not required	1:500
GFP	Chicken	abcam	ab13970	Not required	1:500
Calbindin	Goat	Nittobo	MSFR100400	Not required	1:2000
BLBP	Rabbit	Millipore	ABN14	Citrate	1:300
Nestin	Chicken	Aves lab	NES	Not required	1:1000
Ki67	Rabbit	Thermo Fisher	FM-9106-S1	Citrate	1:200
BrdU	Mouse	BD biosciences	347580	Citrate	1:200
SHH	Rabbit	Santa Cruz	sc9024	Citrate	1:100
RORα	Rabbit	Santa Cruz	sc28612	Citrate	1:200

B. Secondary antibody

Antibody	Source	Catalog Number	Concentration used
Alexa Fluor 488 donkey anti-rabbit IgG	Invitrogen	A21206	1:1000
Alexa Fluor 488 donkey anti-chicken IgY	Jackson Immuno Research	703-545-155	1:1000
Alexa Fluor 555 donkey anti-goat IgG	Invitrogen	A21432	1:1000
Alexa Fluor 555 donkey anti-rabbit IgG	Invitrogen	A31572	1:1000
Alexa Fluor 555 donkey anti-mouse IgG	Invitrogen	A31570	1:1000
Alexa Fluor 594 donkey anti-chicken IgY	Jackson Immuno Research	703-585-155	1:1000
Alexa Fluor 647 donkey anti-goat IgG	Jackson Immuno Research	705-605-003	1:1000

Table S4. Sequences of split-probe sets used for HCR detection of *Shh* and *Gli1*, related to STAR methods.

Target	Refseq accession #	amplifier type	probe1	probe2
Shh	NM_009170	S72	cggtggagtaagaaaacatctggccagcagcagcag	ccagcagcgaggaagcaaggatcacaaggcaagtaggat
		S72	cggtggagtaactgcttgtaggctaaaggggtcagc	ggtcttctcggctacgttgggaataaaggcaagtaggat
		S72	cggtggagtaacttgtgatcttcccttcatatctgc	gtgagttccttaaatcgttcggagtaaggcaagtaggat
		S72	cggtggagtaagtctgctcccgtgttttcctcatcc	gtctttgcacctctgagtcatcagcaaggcaagtaggat
		S72	cggtggagtaaggttcatcacagagatggccaaggc	tcactcgcagcttcactccaggccaaaggcaagtaggat
		S72	cggtggagtaatgaatgatggccgtcctcatcccag	tcgaccctcatagtgtagagactccaaggcaagtaggat
		S72	cggtggagtaaggtcccggtcggacgtggtgatgtc	ggcgagccagcatgccgtacttgctaaggcaagtaggat
		S72	cggtggagtaacatagtagacccagtcgaaacctgc	cagaacagtggatgtgagctttggaaaggcaagtaggat
		S72	cggtggagtaagccggatttggccgccacggagttc	cacggtggcggatcccgggaaacagaaggcaagtaggat
		S72	cggtggagtaacttcaccagcttggtgccgccctgc	cagcacgcggtctccgggacgtaagaaggcaagtaggat
		S72	cggtggagtaagtccaggaaggtgaggaagtcgctg	gaagaccttcttggcgccttcgtcgaaggcaagtaggat
		S72	cggtggagtaacggttgatgagaatggtgccgtgcg	atgacagcgtagcacgaggcgagcaaaggcaagtaggat
		S72	cggtggagtaagagtaccagtggatgcccgcagtcg	caggtgccaatgtggtagagcagctaaggcaagtaggat
		S72	cggtggagtaagggatgcatggtctcgctgtccaac	tcagctggacttgaccgccattcccaaggcaagtaggat
Gli1	NM_010296	S72	cggtggagtaaagaaagtccttctgttcccatgctg	gttggcttggtggcaaaagggcagaaaggcaagtaggat
		S72	cggtggagtaatggtctctctggctgctccataacc	gaaagagagatccttcagtgcagctaaggcaagtaggat
		S72	cggtggagtaatgggtgaggtccggattacggtttg	gagagttgatgaaagccaccagggaaaggcaagtaggat
		S72	cggtggagtaatggtacacagggctggactccatag	catcattgaaccccgagtagagtcaaaggcaagtaggat
		S72	cggtggagtaagcacttgccaaccatcatatccagc	catgtccccttccaaagggtcctccaaggcaagtaggat
		S72	cggtggagtaaacgtgcacttgtgtggcttctcgcc	gtgaataggacttccgacagccttcaaggcaagtaggat
		S72	cggtggagtaagtaaggcttctcacccgtgtgcgac	cttgctgcaaccttcttgctcacacaaggcaagtaggat
		S72	cggtggagtaaggagtgggtccgattctggtgcttg	gagcttgcacacgtatggcttctcaaaggcaagtaggat
		S72	cggtggagtaacaggtagtgacgatgccccattctc	gcatgtagtgctgagcaggtgtgagaaggcaagtaggat
		S72	cggtggagtaagaacagaaagacctcccatccgatc	ggtactcggttcggcttctccaaggaaggcaagtaggat
		S72	cggtggagtaacaggctcttgaaccgctggactctg	cacactagggggcgtgtggacacataaggcaagtaggat
		S72	cggtggagtaatgtatgggttcagaccattgcccat	ccagagtatcagtggaggaaaaatcaaggcaagtaggat
		S72	cggtggagtaagataggagacctgctgggcacagtg	caccccagttttctggggtgggatcaaggcaagtaggat
		S72	cggtggagtaaccggagccttattgctagggtacac	gtcgaggacactggctataggcagcaaggcaagtaggat
		S72	cggtggagtaatgggttctgttgagagataaccatg	aggaggggttgaaattgaggccaagaaggcaagtaggat
		S72	cggtggagtaaattacacaccagctgagctttgagc	caacaattcctgctgcgactgaacgaaggcaagtaggat
		S72	cggtggagtaaggtgcaaagccagatccatatgctg	gagcctgatttgtgattggccgaagaaggcaagtaggat
		S72	cggtggagtaagcgttacacacctgcccttcaggag	gtgttgtccaggtcaagagagtccaaaggcaagtaggat

Table S5. Statistical details of figures, related to STAR methods.

Figure	Statistical values
Fig. 1F	$t(9.83) = 0.54$, $P = 0.6012$
Fig. 1G	$t(11.6) = 1.934$, $P = 0.0778$
Fig. 1H	$t(10.21) = 4.716$, $P = 0.0008$
Fig. 1J	Repeated measure two-way ANOVA: main effect of genotype: $F(1, 12) = 252.7$, $P < 0.0001$, lobule: $F(2.144, 25.73) = 87.21$, $P < 0.0001$, genotype × lobule: $F(2.144, 25.73) = 44.53$, $P < 0.0001$. Post hoc Sidák's multiple comparisons test for effect of genotype on lobule III: $t(5.00) = 4.63$, $P = 0.0226$, VI/VII: $t(5.04) = 10.56$, $P = 0.0005$, IX: $t(6.51) = 4.32$, $P = 0.0165$, X: $t(8.70) = 9.34$, $P < 0.0001$.
Fig. 1N	$t(10.99) = 1.83$, $P = 0.0945$
Fig. 1O	$t(9.18) = 0.3482$, $P = 0.7356$
Fig. 1P	$t(5.088) = 3.154$, $P = 0.0247$
Fig. 1R	Repeated measure two-way ANOVA: main effect of genotype: $F(1, 11) = 147.2$, $P < 0.0001$, lobule: $F(1.841, 20.25) = 27.58$, $P < 0.0001$, genotype × lobule: $F(1.841, 20.25) = 22.75$, $P < 0.0001$. Post hoc Sidák's multiple comparisons test for effect of genotype on lobule III: $t(6.00) = 2.438$, $P = 0.1875$, VI/VII: $t(6.00) = 6.965$, $P = 0.0017$, IX: $t(9.72) = 2.784$, $P = 0.0769$, X: $t(10.95) = 7.682$, $P < 0.0001$.
Fig. 2F	$t(10.59) = 1.763$, $P = 0.1066$
Fig. 2G	$t(10.18) = 0.9470$, $P = 0.3656$
Fig. 2I	$t(13.40) = 0.8189$, $P = 0.4272$
Fig. 3F	One-way ANOVA: $F(2, 13) = 48.51$, $P < 0.0001$. Tukey's post hoc tests: $rl^{+/-}$ vs. $rl^{-/-}$ PC area: $q(13) = 0.87$, $P = 0.8161$; $rl^{+/-}$ vs. $rl^{-/-}$ no PC area: $q(13) = 11.99$, $P < 0.0001$; $rl^{-/-}$ PC area vs. $rl^{-/-}$ no PC area: $q(13) = 12.31$, $P < 0.0001$.
Fig. 3G	One-way ANOVA: $F(2, 13) = 41.54$, $P < 0.0001$. Tukey's post hoc tests: $rl^{+/-}$ vs. $rl^{-/-}$ PC area: $q(13) = 0.1207$, $P = 0.9960$; $rl^{+/-}$ vs. $rl^{-/-}$ no PC area: $q(13) = 11.54$, $P < 0.0001$; $rl^{-/-}$ PC area vs. $rl^{-/-}$ no PC area: $q(13) = 10.93$, $P < 0.0001$.
Fig. 3K	$t(10.96) = 4.239$, $P = 0.0014$
Fig. 4B	One-way ANOVA: $F(2, 11) = 228.4$, $P < 0.0001$. Tukey's post hoc tests: *Dab1* $^{+/fl}$ L7 vs. *Dab1* $^{fl/fl}$ L7: $q(11) = 23.62$, $P < 0.0001$; *Dab1* $^{+/fl}$ L7 vs. *Dab1* $^{fl/fl}$ L7 &SHH: $q(11) = 27.77$, $P < 0.0001$; *Dab1* $^{fl/fl}$ L7 vs. *Dab1* $^{fl/fl}$ L7 &SHH: $q(11) = 2.561$, $P = 0.211$.
Fig. 4D	One-way ANOVA: $F(2, 11) = 10.90$, $P < 0.0025$. Tukey's post hoc tests: *Dab1* $^{+/fl}$ L7 vs. *Dab1* $^{fl/fl}$ L7: $q(11) = 6.081$, $P = 0.0033$; *Dab1* $^{+/fl}$ L7 vs. *Dab1* $^{fl/fl}$ L7 &SHH: $q(11) = 0.5973$, $P = 0.9072$; *Dab1* $^{fl/fl}$ L7 vs. *Dab1* $^{fl/fl}$ L7 &SHH: $q(11) = 5.518$, $P = 0.0064$.
Fig. 5C	$t(5.09) = 0.0922$, $P = 0.93$
Fig. 5D	$t(6.998) = 0.5716$, $P = 0.5855$
Fig. 5E	$t(15.86) = 0.4219$, $P = 0.6788$

Fig. 6F	Two-way ANOVA: interaction, F(6, 57) = 9.233, *P* < 0.0001, stage, F(3, 57) = 19.72, *P* < 0.0001, lobule, F(2, 57) = 12.20, *P* < 0.0001.<br>Post hoc Tukey's multiple comparisons test (within each stage):<br>P6, lobule III vs. VI/VII, *P* = 0.0319; VI/VII vs. IX, *P* = 0.0052.<br>P8, lobule III vs. VI/VII, *P* < 0.0001; VI/VII vs. IX, *P* < 0.0001.
Fig. 6G	Two-way ANOVA: interaction, F(6, 57) = 2.706, *P* = 0.0221, stage, F(3, 57) = 1.207, P = 0.3156, lobule, F(2, 57) = 22.59, *P* < 0.0001.<br>Post hoc Tukey's multiple comparisons test (within each stage):<br>P6, lobule III vs. VI/VII, *P* = 0.0010; VI/VII vs. IX, *P* = 0.0018.<br>P8, lobule III vs. VI/VII, *P* < 0.0001; VI/VII vs. IX, *P* < 0.0001.
Fig. 6H	Two-way ANOVA: interaction, F(6, 57) = 10.53, *P* = 0.0001, stage, F(3, 57) = 35.96, *P* < 0.0001, lobule, F(2, 57) = 1.162, *P* = 0.3202.<br>Post hoc Tukey's multiple comparisons test (within each stage):<br>P2, lobule III vs. VI/VII, *P* = 0.0014; VI/VII vs. IX, *P* = 0.0119.<br>P8, lobule III vs. VI/VII, *P* < 0.0001; VI/VII vs. IX, *P* < 0.0001.
Fig. 7C	*t*(4.318) = 10.15, *P* = 0.0004
Fig. 7D	*U* = 8, *P* = 0.2468
Fig. 7E	*U* = 2, *P* = 0.0173
fig. S1E	*t*(9.286) = 4.982, *P* = 0.0007
fig. S1G	Repeated measure two-way ANOVA: main effect of genotype: F(1, 14) = 71.69, *P* < 0.0001, lobule: F(2.052, 28.72) = 95.63, *P* < 0.0001, genotype × lobule: F(2.052, 28.72) = 58.07, *P* < 0.0001. Post hoc Sidák's multiple comparisons test for effect of genotype on lobule III: *t*(6.082) = 4.437, *P* = 0.0169, VI/VII: *t*(6.114) = 10.57, *P* = 0.0001, IX: *t*(6.744) = 3.149, *P* = 0.0663, X: *t*(8.693) = 7.332, *P* < 0.0002.
fig. S2D	*t*(9.999) = 1.684, *P* = 0.1231
fig. S2E	*t*(7.187) = 1.273, *P* = 0.2425
fig. S2F	*t*(9.410) = 1.802, *P* = 0.1036
